# Sandwich d/f Heterometallic Complexes [(Ln(hfac)_3_)_2_M(acac)_3_] (Ln = La, Pr, Sm, Dy and M = Co; Ln = La and M = Ru)

**DOI:** 10.3390/molecules29163927

**Published:** 2024-08-20

**Authors:** Cristian Grechi, Silvia Carlotto, Massimo Guelfi, Simona Samaritani, Lidia Armelao, Luca Labella

**Affiliations:** 1 Dipartimento di Chimica e Chimica Industriale and CIRCC, Università di Pisa, via Giuseppe Moruzzi 13, 56124 Pisa, Italy; cristian.grechi@libero.it (C.G.); massimo.guelfi@unipi.it (M.G.); simona.samaritani@unipi.it (S.S.); 2 Istituto di Chimica della Materia Condensata e di Tecnologie per l’Energia (ICMATE), Consiglio Nazionale delle Ricerche (CNR) e INSTM, Presso Dipartimento di Scienze Chimiche, Università di Padova, Via Marzolo 1, 35131 Padova, Italy; silvia.carlotto@unipd.it; 3 Dipartimento di Scienze Chimiche e INSTM, Università di Padova, Via Marzolo 1, 35131 Padova, Italy; lidia.armelao@unipd.it; 4 Centro per l’Integrazione della Strumentazione Scientifica dell’Università di Pisa (C.I.S.U.P.), Università di Pisa, 56126 Pisa, Italy; 5 Dipartimento di Scienze Chimiche e Tecnologie dei Materiali (DSCTM), Consiglio Nazionale delle Ricerche (CNR), Piazzale A. Moro 7, 00185 Roma, Italy

**Keywords:** heterometallic complexes, lanthanides, cobalt, ruthenium, quantum mechanical calculations

## Abstract

Sandwich d/f heterometallic complexes [(Ln(hfac)_3_)_2_M(acac)_3_] (Ln = La, Pr, Sm, Dy and M = Co; Ln = La and M = Ru) were prepared in strictly anhydrous conditions reacting the formally unsaturated fragment [Ln(hfac)_3_] and [M(acac)_3_] in a 2-to-1 molar ratio. These heterometallic complexes are highly sensitive to moisture. Spectroscopic observation revealed that on hydrolysis, these compounds yield dinuclear heterometallic compounds [Ln(hfac)_3_M(acac)_3_], prepared here for comparison purposes only. Quantum mechanical calculations supported, on the one hand, the hypothesis on the geometrical arrangement obtained from ATR-IR and NMR spectra and, on the other hand, helped to rationalize the spontaneous hydrolysis reaction.

## 1. Introduction 

The interest in heterometallic complexes with *3d* and *4f* metal ions is rapidly growing because of their various returns in the fields of catalysis [1], the synthesis of binary oxide nanomaterials [2], medicine [3,4,5], luminescence [6,7] and molecular magnetism [8,9,10].

**Scheme 1:**
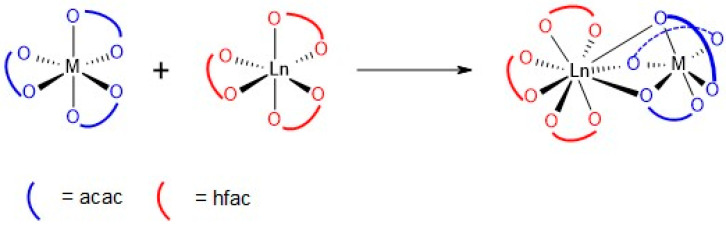
Heterodinuclear metal complex formation, exemplified here for a formally unsaturated [Ln(hfac)_3_] and [M(acac)_3_] with the metal M in an octahedral *tris*-chelate environment.

The rational design of 3d–4f heterometallic complexes is hampered by the complexity of lanthanide coordination chemistry characterized by high lability and the tendency to high and variable coordination numbers. The rich structural diversity of heterometallic complexes is partly due to the often-adopted serendipitous self-assembly synthetic procedure. To avoid/limit the formation of mixtures of products, great attention normally has to be paid to the nature of the used ligands. Since 3d and 4f metal centers show largely different affinities towards different donor atoms (such as nitrogen and oxygen), it is possible to design multi-site coordination ligands with appropriate donor sets able to discriminate different metal centers and a bridging ligand able to keep the different metal fragments linked in the same molecular architecture [11,12,13,14]. Synthetic control is often improved using the metallo-ligand strategy, where a metal complex (normally a d-transition [15,16,17], less commonly a f-transition complex [18,19,20,21,22,23,24,25,26]) can behave as a ligand for another metal in a smooth reaction in mild conditions. The “complex as ligand” strategy, can afford heterometallic complexes, where the coordination sphere of the lanthanide ion can be normally predicted. An attractive synthetic path to heterometallic complexes exploits a Lewis base-to-Lewis acid interaction of a molecular d or p complex with a formally unsaturated *tris*-diketonato lanthanide fragment [27,28,29,30,31,32,33,34,35]. This simple route relies on the strong preference of a diketonato ligand for a chelate coordination mode and on the tendency of a [Ln(dike)_3_] unit to react with a neutral Lewis base, forming mononuclear complexes with typical coordination numbers 8 or 9. Hexafluoroacetylacetonato (hfac) ligands, presenting a high electron-withdrawing ability, have been mostly used since they favor reactions even with low-basicity donors. This synthetic approach to stable heterometallic complexes, initially most commonly used for Shiff-based [36,37,38,39,40,41] copper complexes [42,43], can be extended to other Lewis bases, at a variance of the metal and its coordination sphere with the aims of tuning the properties of the Lewis base and understanding the structure−property relationships for these molecular architectures with two cations in close proximity to monoatomic oxo bridges. Facially coordinated [LnM(hfac)_3_(µ_2_-acac-O,O,O)_3_] (Ln = La, Pr, Gd; M = Cr, Fe, Ga) heterodinuclear systems have been reported in the literature along with interesting magnetic studies [44]. Moreover, following this synthetic approach, heterometallic complexes [Ln(hfac)_3_Al(LL)_3_] (with Ln = Eu, Tb, Gd and HLL’ = methyl acetoacetate, salicylaldehyde, 2-hydroxynaphthaldehyde and Ln = Eu, Gd, Er and HLL’= 8-hydroxyquinoline *N*-oxide) with tunable luminescence properties have been prepared [45,46]. X-ray diffraction studies have clarified that all compounds are isostructural heterodinuclear complexes, where the coordination geometry of the lanthanide center is a tricapped trigonal prism that shares one face of the octahedron of the tripositive d or p center. The only exception is related to the smaller erbium compound with HLL’ = 8-hydroxyquinoline *N*-oxide, where only two oxygen atoms bridge the two metal atoms in the dinuclear molecular architecture [46]. In summary, the procedure reported in Scheme 1, affording dinuclear heterometallic complexes, is potentially applicable across the lanthanide series with different tripositive metal complexes.

**Scheme 2:**
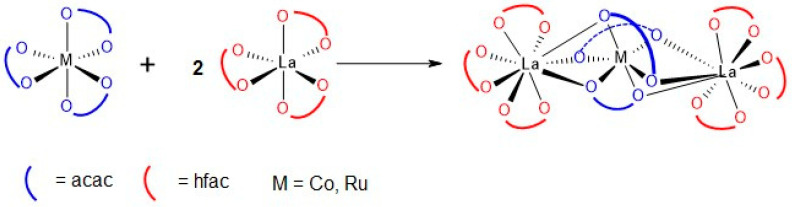
Heterotrinuclear f-d-f sandwich metal complex formation, exemplified here for Ln = La and M = Co or Ru in an octahedral *tris*-chelate environment.

In this class of complexes, if an asymmetric chelate is used, the *fac* isomer is always involved in the coordination to the lanthanide, with the higher basicity donor oxygen atom in a bridging coordination mode. If a symmetric chelate is used, in principle, the two opposite octahedral faces should be equivalent with respect to the coordination of a lanthanide fragment. In this work, we show that Lewis acid–Lewis base adducts can also be prepared with a different stoichiometry and that the mononuclear octahedral Lewis base [M(acac)_3_] can interact with two Lewis acid fragments. Larger lanthanides centers have been chosen in this work based on the idea that an enhanced tendency to higher coordination numbers could promote a strong bonding interaction, while an inert trivalent d metal center could facilitate the work-up and characterization of the products. Sandwich-type complexes f-d-f [(Ln(hfac)_3_)_2_M(acac)_3_] (Scheme 2: Ln = La, Pr, Sm, Dy and M = Co; Ln = La and M = Ru) were obtained following the same synthetic approach used for heterodinuclear d/f compounds that were prepared here for comparison purposes.

## 2. Results and Discussion

### 2.1. Synthesis of Heterodinuclear Complexes

**Figure 1:**
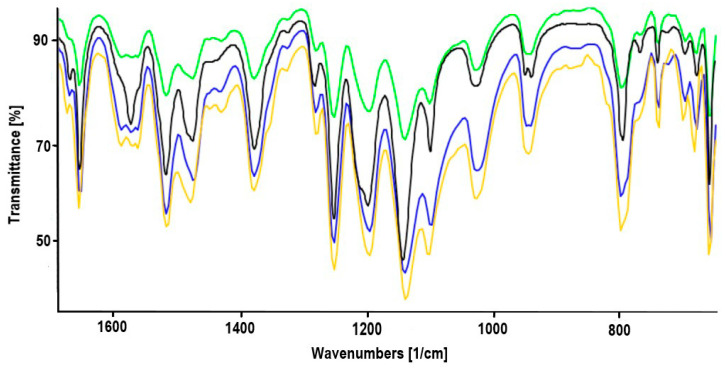
ATR-IR spectra of heterodinuclear [Ln(hfac)_3_Co(acac)_3_] (**1Ln**) complexes: Ln = La, (black); Pr, (blue); Sm, (green) and Dy (orange).

Formally coordinatively unsaturated complexes [Ln(hfac)_3_] (Ln = La, Pr, Sm and Dy) react in anhydrous conditions in a non-polar solvent with an equimolar amount of [Co(acac)_3_]. The reaction can be monitored following the dissolution of the initially sparingly soluble lanthanide precursor. A dark green solution is normally obtained by heating the initial mixture at 60–80 °C for about half an hour. No side product was observed. The well-soluble complexes can be recovered as a crude product in good yield by simply removing in vacuo the volatiles phases, and they can be recrystallized from hot heptane. The analytical data are consistent with heterodinuclear complexes [Ln(hfac)_3_M(acac)_3_], **1Ln**, and they show very similar ATR-IR spectra (Figure 1). Almost perfectly superimposable IR spectra suggest a similar molecular architecture for all new complexes.

All products are air-stable in the solid state. The ATR-IR spectra registered after short-term exposures to air do not show changes in the pattern.

**Figure 2:**
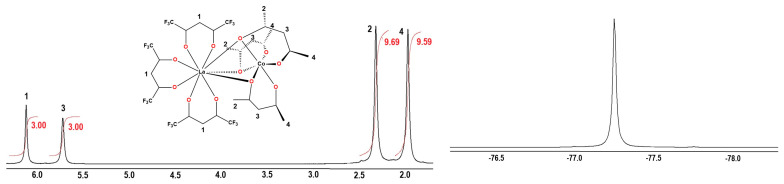
The ^1^H (**left**) and ^19^F (**right**) NMR of [La(hfac)_3_Co(acac)_3_], **1La**.

The diamagnetic lanthanum complex **1La** (Figure 2) shows that this acid-to-base interaction is stable in solution and in the solid state. Diketonato C-H of the two building blocks (lanthanum and cobalt) integrate in a ratio of 1 to 1, and methyl groups of acac ligands are differentiated by coordination of only one oxygen atom for each acac to lanthanum so that two singlets with the same integral are present. Consistently a single signal at −77.24 ppm was observed in the ^19^F NMR of the **1La** compound.

NMR details (chemical shifts and ratios of the integrals) of this new compound strongly reflect those of the analog [Eu(hfac)_3_Al(meac)_3_], whose structure has been obtained through single crystal X-ray diffraction [45]. Consistently, the ^1^H and ^19^F NMR spectra of **1Pr** and **1Sm** (Figures S1 and S2) show the presence of a single product in solution, presenting the two metal fragments in the expected molar ratio. In both ^1^H NMR spectra, the acetylacetonato methyl groups are not equivalent (this can be noticed also in Figure S3 in the spectrum of the ruthenium analog, see below). These data suggest a similar molecular structure for these **1Ln** compounds. An X-ray diffraction study on **1Sm** confirmed the molecular identity (Figure S6). The octahedral cobalt compound shares a trigonal face with the tricapped trigonal prism of a nona-coordinated samarium center (Figures S7 and S8). Three bridging oxygen atoms of three different acac ligands complete the lanthanide coordination sphere beside three chelating hfac ligands. Although, as expected, **1Sm** was isostructural with several heterodinuclear d/f compounds previously reported in the literature [44], the adopted crystalline phase is different since, as already noticed [44], this class of compounds is inclined to polymorphism. In the crystal structure of **1Sm**, two independent molecules are present in a centrosymmetric P21/c space group (Table S1). Selected bond angles and bond distances are reported in Tables S2 and S3.

### 2.2. Synthesis of Heterotrinuclear Sandwich Complexes

**Figure 3:**
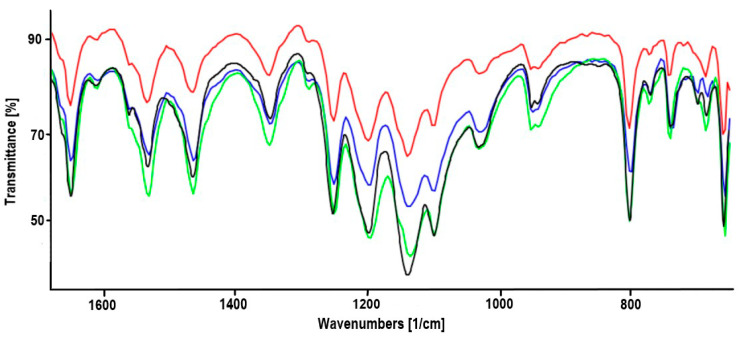
ATR-IR spectra of [(Ln(hfac)_3_)_2_Co(acac)_3_] (**2Ln**): La, (black); Pr, (blue); Sm, (green), Dy, (red).

Heterodinuclear complexes were obtained with a ratio of 1:1 between the two reagents. In a synthesis carried out with a slight excess of the lanthanide fragment, we expected to have to filter off the excess of the sparingly soluble lanthanide precursor. However, the reproducible formation of a solution suggested the possibility of a different stoichiometry of the reaction. A molar ratio of two between the lanthanide and cobalt building blocks was established since any excess of the scarcely soluble lanthanide precursor with respect to this molar ratio was found unreacted. The optimized synthetic protocol suggests the use of an excess of the lanthanide precursor in a reaction in strictly anhydrous conditions in dichloroethane (DCE) at 60 °C for 30′. After cooling to room temperature, a suspension was obtained with a small amount of the lanthanide precursor, which was filtered from the green solution. Removing the volatiles in vacuo, a green residue was obtained that was maintained over P_4_O_10_ for a few hours. Products can be crystallized from hot toluene, yielding green crystals of the products in good yields. These products are highly soluble in chlorinated solvents but have limited solubility, largely increasing with temperature, in toluene. Elemental analyses support a composition [(Ln(hfac)_3_)_2_Co(acac)_3_] for Ln = La, Pr, Sm and Dy (**2Ln**). The ATR-IR spectra of these compounds are all very similar, as shown in Figure 3.

**Figure 4:**
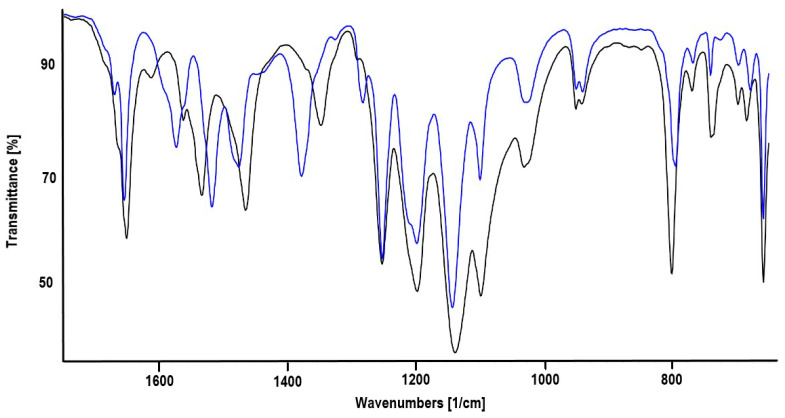
ATR-IR spectra of [(La(hfac)_3_)_2_Co(acac)_3_] (black) and [La(hfac)_3_Co(acac)_3_] (blue).

In Figure 4, the ATR-IR spectra of the two heterometallic complexes [(La(hfac)_3_)_2_Co(acac)_3_] and [La(hfac)_3_Co(acac)_3_] are compared as examples of the two families of compounds.

**Figure 5:**
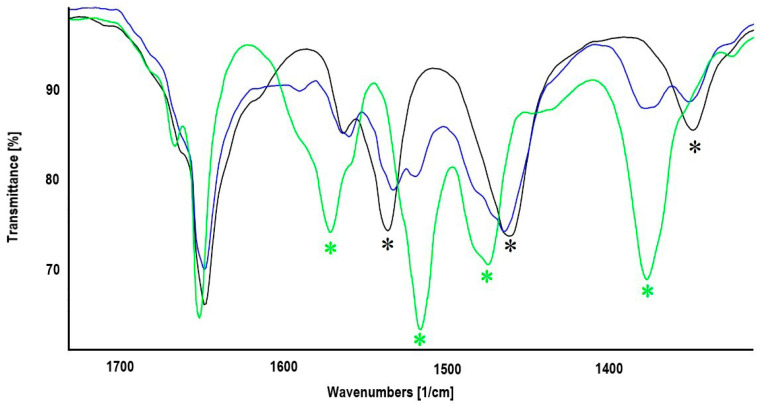
ATR-IR in the range of 1700–1350 cm^−1^: (i) [(La(hfac)_3_)_2_Co(acac)_3_] (black); (ii) [(La(hfac)_3_)_2_Co(acac)_3_] after 1 h air exposure (blue); (iii) [La(hfac)_3_Co(acac)_3_] (green). Bands increasing (green *) or decreasing (black *) with time are marked.

In more detail, it is possible to note that the two spectra are almost superimposable in the range between 1300 and 1100 cm^−1^, typical for C-F vibrations associated with a Ln(hfac)_3_ fragment [47,48]. These bands do not change with air exposure, suggesting that for the two compounds, few changes occur in the hfac ligand in the lanthanide fragment. On the other hand, it is possible to note differences in the range between 1600 and 1350 cm^−1^, possibly associated with acetylacetonato ligands. The spectra of the trimetallic complexes [(Ln(hfac)_3_)_2_Co(acac)_3_] show a marked air sensitivity also in the solid state, and ATR-IR spectra in the range between 1600 and 1300 cm^−1^ change with time on air exposures. In Figure 5, the change in ATR-IR with time for the heterotrinuclear compound suggests the possible formation of dinuclear [La(hfac)_3_Co(acac)_3_] as the hydrolysis product. In Figure 5, the bands that are gradually disappearing (1608, 1530, 1462 and 1345 cm^−1^) and the new bands (1569, 1514, 1473 and 1375 cm^−1^) are marked to clarify this point.

A similar behavior was noticed for all trimetallic ATR-IR spectra. The major difference concerned the hydrolysis rate that qualitatively was observed to increase moving to the right across the lanthanide series.

**Figure 6:**
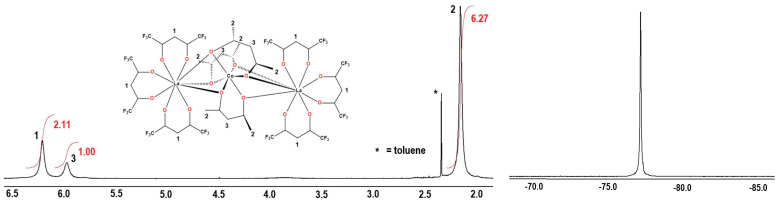
The ^1^H (**left**) and ^19^F (**right**) NMR spectra of [(La(hfac)_3_)_2_Co(acac)_3_] in CDCl_3_.

**Figure 7:**
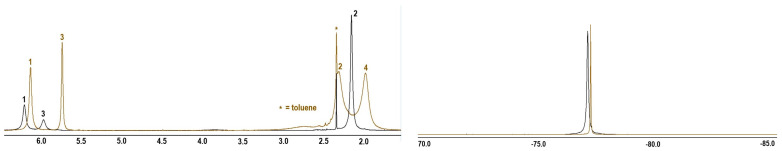
The ^1^H and ^19^F NMR spectra of [(La(hfac)_3_)_2_Co(acac)_3_] in CDCl_3_: (i) immediately after preparation (black) and (ii) after a few days (brown).

The diamagnetic [(La(hfac)_3_)_2_Co(acac)_3_] afforded an NMR characterization, significantly showing a single signal for the methyl groups in the ^1^H NMR (see Figure 6) that were not equivalent in the spectrum of the heterodinuclear complexes (see Figure 2). For [La(hfac)_3_Co(acac)_3_], the methyl close to the coordinated carbonyl group is different from that close to the carbonyl group not interested in the coordination. In [(La(hfac)_3_)_2_Co(acac)_3_], both carbonyl groups are coordinated symmetrically, and a single signal in the ^19^F NMR is also present. Also, in solution, hydrolysis was observed with time. After a few days, the NMR spectra of the same sample became compatible with those of the heterodinuclear [La(hfac)_3_Co(acac)_3_] compound, and the formation of a colorless solid, identified through ATR-IR spectroscopy as [La(hfac)_3_(H_2_O)_3_], was noticed. Consistently, an authentic sample of the latter compound showed a scarce solubility in CDCl_3_ and signals at 6.21 ppm in ^1^H NMR and at −76.8 ppm in ^19^F NMR; unobserved in the NMR spectra (Figure 7) since they are covered by the intense signals of the well-soluble heterodinuclear compound [La(hfac)_3_Co(acac)_3_].

Similarly, it was noticed that all **2Ln** compounds are highly susceptible to hydrolysis in solution, and the spectra are complicated by the presence of the hydrolysis products. As an example, the ^1^H NMR of **2Sm** is shown in Figure S4, where the composition of the mixture significantly changes after 30′ even without any deliberate exposure of the sample to air. These spectra attest to the presence of **2Sm** in solution and its conversion into **1Sm** and [Sm(hfac)_3_(H_2_O)_2_] (Figure S5) for hydrolysis.

Despite several attempts, a full characterization through single-crystal X-ray diffraction of these heterotrinuclear compounds was not achieved, possibly because of (i) the low quality of the single crystals and/or (ii) high air sensitivity. However, reproducibly, single crystals for **2Pr** and **2Sm** showed the same rhombohedral unit cell. Although the quality of data did not reach the standards for publication, X-ray diffraction experiments suggested a linear arrangement of the three metal atoms, with [Co(acac)_3_] bridging two [Ln(hfac)_3_] fragments and behaving as a tridentate ligand for each lanthanide atom. This symmetrical environment, in agreement with the NMR spectra, suggests a sandwich structure where the two opposite triangular faces of octahedral [Co(acac)_3_] behave as Lewis bases towards two different [Ln(hfac)_3_] fragments. A theoretical study to support the validity of this structural hypothesis was carried out.

### 2.3. Quantum Mechanical Calculations

To gain additional information on the stability of the different heteronuclear complexes, [Ln(hfac)_3_Co(acac)_3_] and [(Ln(hfac)_3_)_2_Co(acac)_3_], quantum mechanical calculations were performed with PBE functional, def2/JK auxiliary basis set, and the inclusion of dispersion correction by adopting Grimme’s DFT-D3. Figure S9 reports the [La(hfac)_3_Co(acac)_3_] optimized structure and the similar Pr and Sm complexes. Relevant structural parameters such as Ln‧‧‧Co, Ln-O_bridge_ and Co-O_bridge_ are listed in Table 1.

Dependent the reduction on the ionic radius for Ln^3+^ across the series, bond-length reductions are evident for Ln-O bonds in both heterodinuclear and trinuclear compounds [49,50,51]. In addition to the bond lengths, another parameter that can provide information about the strength of the bond is the Mayer bond order. Indeed, the variation in the bond strengths across the series can be quantitatively estimated by comparing their Mayer bond order (MBO) [52]. The MBO parameter indicates the bonding strength of two adjacent atoms that are not necessarily directly connected. The higher the Mayer bond order, the more stable the bond. For the same kind of bond, the following correlation can be obtained with the bond order: the longer the bond length, the lower the bond order. The MBO analysis in Table 1 reveals a marked increase in the MBO values of the heterodinuclear compounds for Ln-O_bridge_ and Co-O_bridge_ bonds moving from La to Pr, while no significant difference is observed from Pr to Sm. Finally, the comparison between the bond lengths and the MBO of [Co(acac)_3_] and the heterodinuclear product [Ln(hfac)_3_Co(acac)_3_] shows longer bond lengths and a smaller MBO going from the cobalt precursor to products (Table 1).

**Figure 8:**
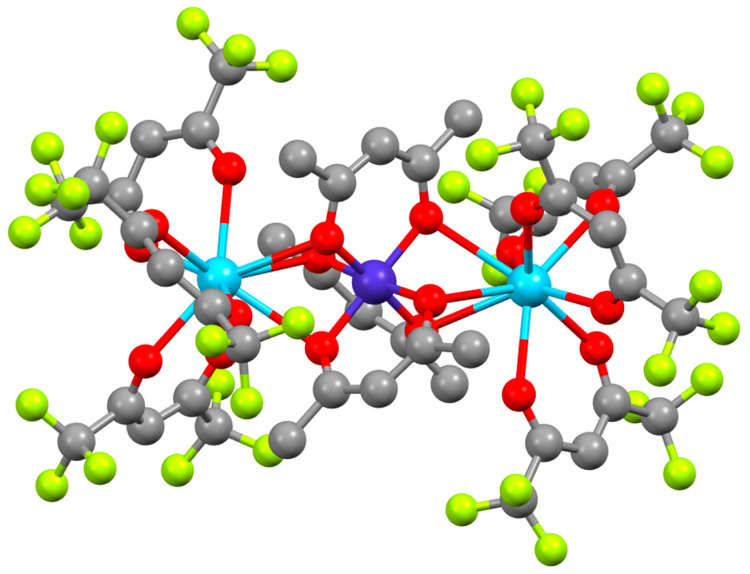
Optimized structure of [(La(hfac)_3_)_2_Co(acac)_3_] (**2La**). The green, grey, cyan, red and violet spheres are F, C, La, O and Co atoms, respectively. H atoms are omitted for clarity. Level of theory: PBE def2/JK, DFT-D3.

Quantum mechanical calculations were also performed on the heterotrinuclear complexes. The optimized structures for all heterotrinuclear compounds, see Figure 8 for [(La(hfac)_3_)_2_Co(acac)_3_] (**2La**) and Figure S10 for Pr and Sm, confirm the coordination proposed by spectroscopic data.

In the optimized structure, each chelate acetylacetonato ligand on an octahedral cobalt also acts as a bridging ligand, bonding with two different oxygen atoms and two different lanthanum metals. This arrangement results in a symmetrical structure where each lanthanum is nona-coordinated within a tricapped trigonal prism geometry, sharing a face of the cobalt octahedron. In this structure, all methyl groups are equivalent, as also found in the solution ^1^H NMR. An inspection of the geometrical parameters in Table 1 for heterotrinuclear compounds shows that the Co-O_bridge_ bond lengths in the heterotrinuclear complexes are almost equal to the reagent [Co(acac)_3_], in contrast to the heterodinuclear complexes, where a more significant elongation is observed. In both the heterodinuclear and heterotrinuclear complexes, the MBO indexes decrease, but this reduction is more significant for the former.

To further support the structural hypothesis, a comparison between the experimental and simulated IR spectra was performed for both the lanthanum heterodinuclear (**1La**) and heterotrinuclear (**2La**) species. The very good agreement between the experimental and simulated spectra for both complexes (see Figure S12) suggests the correctness of the structural hypothesis. The assignment of the most relevant IR bands to vibrational modes for **1La** and **2La** is reported in Tables S6 and S7 of the ESI. For both complexes, the vibrational modes below 1200 cm^−1^ are assigned to the C-F stretching of the La fragments and are almost invariant. Relevant variations are observed in the range between 1250 and 1550 cm^−1^, where the C=O and C-H stretching on the Co fragments changes their intensities and positions. Relevant vibrational modes above 1600 cm^−1^, assigned to C-O stretching on the La fragments, remain almost invariant. To support the comparison between simulated and experimental spectra for the two complexes, Figure S12 is reported in ESI.

In addition to the geometrical parameters, quantum mechanical calculations allowed us to gain information on the stabilities of the heterodinuclear and heterotrinuclear species across the series. Stability can be calculated by considering the difference in the electronic energies between the optimized product [Ln(hfac)_3_Co(acac)_3_] and the two optimized reactants [Ln(hfac)_3_] and [Co(acac)_3_]. The Gibbs free energy should be rigorously considered for a better stability assessment, but given the similarity in the structures, an almost similar value can be assumed for entropy. For the heterodinuclear species, the electronic energies (∆E) are −45.2, −39.2 and −37.5 kcal/mol for the La, Pr and Sm complexes, respectively (see Table 2). The entropy contribution (−T∆S) is equal to 14.27 kcal/mol and it is calculated, similarly to the other thermodynamic parameters, as the difference between the entropy of the product [La(hfac)_3_Co(acac)_3_] and those of the reactants [Ln(hfac)_3_] and [Co(acac)_3_]. By considering this value for entropy, the Gibbs free energies ∆G are −28.4, −22.5 and −20.8 kcal/mol for the La, Pr and Sm complexes, respectively (see Table 2). These results suggest the following important highlights: (i) based on the Gibbs free energy values, all species are stable, in agreement with experimental data, but (ii) the stability of the heterodinuclear complexes decreases across the series, with a reduction in the ionic radius, where the weaker Lewis acid (La) generates the most stable complex.

Analogously to the heterodinuclear complexes, the stability of the heterotrinuclear derivatives can be evaluated as the difference between the optimized product [Ln((hfac)_3_)_2_Co(acac)_3_] and the two optimized reactants 2[Ln(hfac)_3_] and [Co(acac)_3_]. The only difference in the heterodinuclear complexes is the ratio between reagents [La(hfac)_3_]/[Co(acac)_3_] as follows: one for the dinuclear and two for the trinuclear. The electronic energies are −81.7, −71.5 and −65.6 kcal/mol for the La, Pr and Sm heterotrinuclear complexes, respectively. By considering an entropy contribution equal to 29.8 kcal/mol (calculated for [(La(hfac)_3_)_2_Co(acac)_3_]), the Gibbs free energies are −47.0, −36.8 and −30.9 kcal/mol for the La, Pr and Sm complexes, respectively (see Table 2). Similar to the **1Ln** heterodinuclear complexes, the stability of the **2Ln** trinuclear complexes decreases across the series; then, the La complex is more stable than the Sm complex.

**Figure 9:**
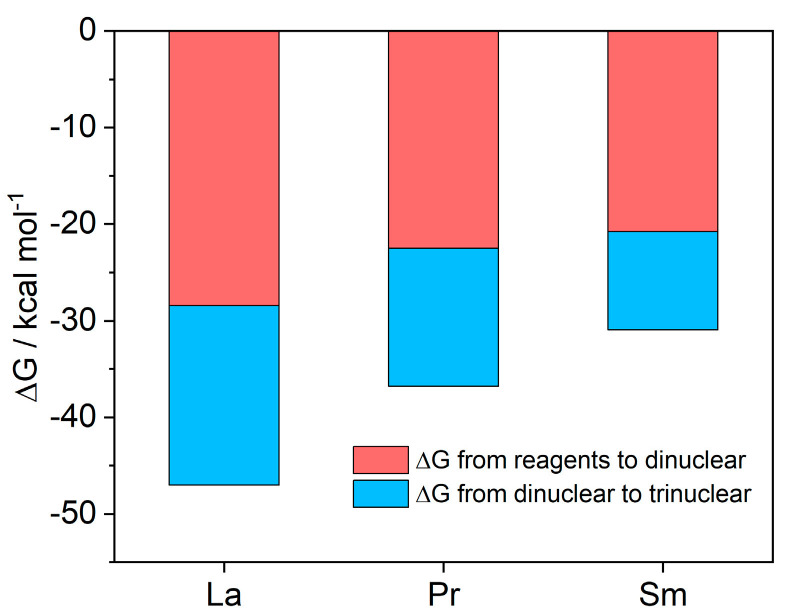
The stability trend, as ∆G values, for the dinuclear [Ln(hfac)_3_Co(acac)_3_] and trinuclear [(Ln(hfac)_3_)_2_Co(acac)_3_] complexes (Ln = La, Pr and Sm). All values are in kcal/mol.

These values refer to the stability of the heterotrinuclear complexes starting from [Co(acac)_3_] and [La(hfac)_3_], in agreement with experimental synthesis. Comparing the stability of the two classes of complexes, the calculations suggest that (Figure 9 and Table 2) a relatively small contribution (blue bars in Figure 9) results in moving from the stability of the heterodinuclear (red bars in Figure 9) to that of the heterotrinuclear compound. These values suggest that (i) the dinuclear species are more stable than the trinuclear species and (ii) the stability of both the dinuclear and trinuclear species decreases across the series with the reduction in the Ln ionic radius.

From a thermodynamic perspective, considering the hydrolysis reaction of [(Ln(hfac)_3_)_2_Co(acac)_3_], which yields [(Ln(hfac)_3_Co(acac)_3_] and [Ln(hfac)_3_(H_2_O)_3_] as products, the calculations revealed an electronic energy change (∆E) of −17.4 kcal/mol and a Gibbs free energy change (∆G) of −1.2 kcal/mol for [(La(hfac)_3_)_2_Co(acac)_3_]. This suggests a spontaneous process. The calculated electronic energies (∆E) for Pr and Sm are −17.8 and −18.3 kcal/mol, respectively. These values imply an increased stability of the hydrolysis products across the series, consistent with the experimentally observed faster hydrolysis process.

### 2.4. Studies on the Lanthanum/Ruthenium Analogs

Given the challenging conditions of our experimental studies, which demanded strict anhydrous conditions and led to sandwich structures that were difficult to characterize and study, we made an effort to stabilize the product. To achieve this, the Lewis base [Ru(acac)_3_] featuring an inert 4*d* metal center was employed along with the Lewis acid [La(hfac)_3_], which had demonstrated favorable outcomes in previous experiments involving [Co(acac)_3_].

**Figure 10:**
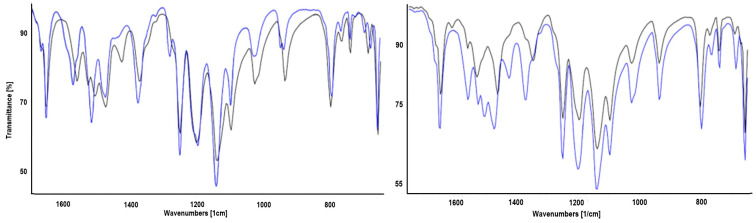
(**Left**): ATR-IR of [La(hfac)_3_Ru(acac)_3_] (black) and [La(hfac)_3_Co(acac)_3_] (blue). (**Right**): ATR-IR of [(La(hfac)_3_)_2_Ru(acac)_3_] (black) and [La(hfac)_3_Ru(acac)_3_] (blue).

Using the same experimental protocol presented above, two heterometallic compounds, i.e., [La(hfac)_3_Ru(acac)_3_] and [(La(hfac)_3_)_2_Ru(acac)_3_], were isolated. First, the spectra of [La(hfac)_3_M(acac)_3_] (M = Co and Ru) are quite similar, as shown in Figure 10 on the left, so it is possible to assume that they have the same molecular identity.

Differences between the two ruthenium complexes in the range 1600–1300 cm^−1^ can be clearly observed, as in the previous study on cobalt complexes (Figure 10, right). Quantum mechanical calculations were performed on Ru complexes. The good agreement also between the experimental and simulated IR spectra for both the heterotrinuclear and heterodinuclear species, as reported in Figure S13 of the ESI, further supports the structural hypothesis.

**Figure 11:**
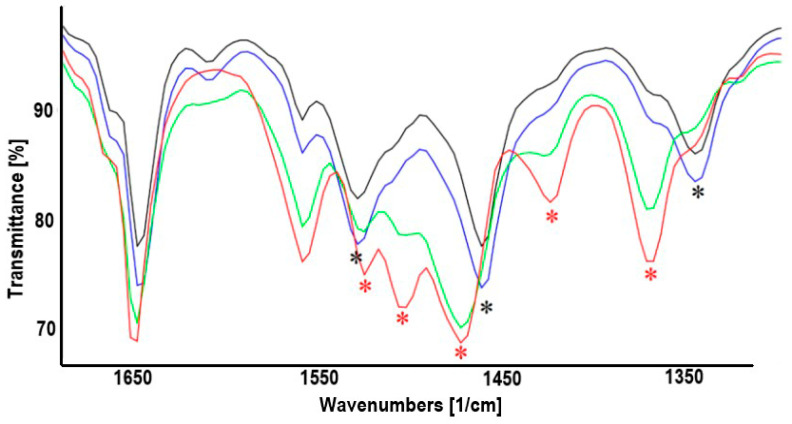
ATR-IR of (i) [(La(hfac)_3_)_2_Ru(acac)_3_] (black); (ii) after 4′ of air exposure (blue); (iii) after 30′ (green) and [La(hfac)_3_Ru(acac)_3_] (red). Bands increasing (red *) or decreasing (black *) with time are marked.

Although the elemental analysis and spectroscopic data indicate the isolation of a sandwich ruthenium lanthanum complex, a strong susceptibility to hydrolysis was also observed in the solid state of this compound. After only a few minutes of air exposure, evident changes in the ATR-IR spectrum suggested the conversion of the product into the corresponding heterodinuclear compound (Figure 11).

To further support the experimental findings, optimized structures (see Figure S11) were also obtained for [La(hfac)_3_Ru(acac)_3_] and [(La(hfac)_3_)_2_Ru(acac)_3_]. The relevant bond lengths and MBO values are reported in Table S4. The calculations show that the Co and Ru structures have a similar geometrical arrangement, and the bond length trends between the heterodinuclear and heterotrinuclear complexes are also similar. In more detail, a progressive reduction for Ru-O_bridge_ is found from the mononuclear to dinuclear and trinuclear structures. The sign and the amount of reduction are similar for the Co and Ru complexes. The stability of the dinuclear Ru species was calculated by considering the difference in the electronic energies between the optimized product, [(La(hfac)_3_Ru(acac)_3_], and the two optimized reactants, [La(hfac)_3_] and [Ru(acac)_3_]. Values of −42.8 and −25.7 kcal/mol were obtained for ∆E and ∆G, respectively (see Table S5). Both these values are lower than those of [La(hfac)_3_Co(acac)_3_], suggesting lower stability of the dinuclear Ru complex. The stability of the trinuclear Ru complex was analogously evaluated as a difference between reagents ([Ru(acac)_3_] and 2[La(hfac)_3_]) and the product [(La(hfac)_3_)_2_Co(acac)_3_], and values of −78.4 and −43.6 kcal/mol were obtained for ∆E and ∆G, respectively (see Table S5). The stabilization from the dinuclear to trinuclear is then −35.7 and −17.9 kcal/mol for ∆E and ∆G, respectively (see Table S5). The following conclusions can be drawn from these values: (i) there is a limited increase in the stability going from the dinuclear to the trinuclear complex; this increase is significantly lower with respect to the formation of the heterodinuclear complex and (ii) the comparison with the analogous dinuclear and trinuclear cobalt complexes shows a generally lower stability of the Ru complexes (lower stability values in Table S5). Finally, for the hydrolysis reaction of [(La(hfac)_3_)_2_Ru(acac)_3_] yielding [(La(hfac)_3_Ru(acac)_3_] and [La(hfac)_3_(H_2_O)_3_], the calculation found an electronic energy ∆E of −18.2 kcal/mol and a ∆G −1.8 kcal/mol, which suggested that the hydrolysis of the Ru trinuclear species is a spontaneous process and that is more favored than for the analogous cobalt compound (∆E = −17.4 and ∆G = −1.2 kcal/mol).

## 3. Materials and Methods

All manipulations were performed under a dinitrogen atmosphere using anhydrous solvents. Anhydrous [Ln(hfac)_3_] species (Ln^3+^= La^3+^, Pr^3+^, Sm^3+^ and Dy^3+^) were obtained by dehydration of the corresponding hydrate complex [Ln(hfac)_3_(H_2_O)_n_] (n = 3, Ln = La; n = 2, Ln = Pr, Sm), according to the procedure reported in the literature [53,54]. FTIR spectra on solid samples were recorded with a Perkin–Elmer “Spectrum One” spectrometer, equipped with an ATR accessory. A Bruker “Avance DRX400” spectrometer was used to record ^1^H and ^19^F NMR spectra. Chemical shifts were measured in ppm (δ) from TMS by residual solvent peaks for ^1^H and from CFCl_3_ for ^19^F. Elemental analysis (C, H, N) was performed with an Elementar “vario MICRO cube” instrument at Dipartimento di Chimica e Chimica Industriale, Università di Pisa.

The synthesis of [Ln(hfac)_3_Co(acac)_3_] (Ln = La, Pr, Sm and Dy) **1Ln** is described below:

The synthesis of the lanthanum derivative [La(hfac)_3_Co(acac)_3_] (**1La**) is described at length; the same experimental protocol was used for the other **1Ln** compounds.

The synthesis of **1La** was performed as follows: To a suspension of [La(hfac)_3_] (0.935 g; 1.23 mmol) in toluene (80 mL), [Co(acac)_3_] (0.430 g; 1.21 mmol) was added. After 30′ stirring at 60 °C, a dark green solution was obtained. After removing the volatiles at reduced pressure, solid a green residue was recovered (1.086 g; 0.97 mmol; 80% yield for [La(hfac)_3_Co(acac)_3_]. The compound can be recrystallized from a cold heptane solution (−20 °C). The elemental analysis calculated for [La(hfac)_3_Co(acac)_3_] (C_30_H_24_CoF_18_LaO_12_) was as follows: C: 32.3% and H: 2.2%; found C: 31.9% and H: 2.0%. ATR-IR (range 1700–650 cm^−1^): 1665 (w), 1650 (s), 1569 (m), 1514 (s), 1473 (m), 1375 (s), 1323 (w), 1280 (m), 1250 (s), 1196 (s), 1141 (s), 1098 (m), 1027 (m), 948 (m), 939 (m), 795 (m), 768 (w), 740 (w), 697 (w), 678 (w) and 658 (m). It is stable for short-term air exposures, as evidenced by the almost over-imposable ATR-IR spectra of the sample in air.

**Figure 12:**
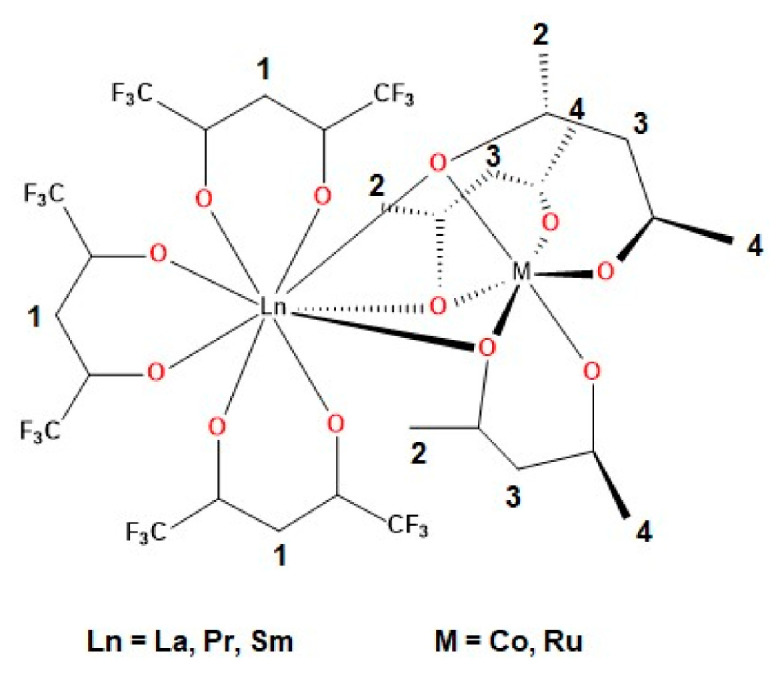
Numbering of ^1^H NMR protons in [Ln(hfac)_3_M(acac)_3_].

The ^1^H NMR (CDCl_3_, ppm) was as follows: 6.13 (s, H^1^, 3H), 5.73 (s, H^3^, 3H), 2.32 (s, H^4^, 9H), 1.97 (s, H^2^, 9H), (Figure 12).

The ^19^F NMR (CDCl_3_, ppm) was as follows: −77.24 (s, 18F).

This compound is highly soluble in toluene and chlorinated solvents, and partially soluble in heptane.

The synthesis of **1Pr** was performed as follows: Starting from [Pr(hfac)_3_] (0.402 g; 0.53 mmol) and [Co(acac)_3_] (0.187 g; 0.53 mmol) in toluene (40 mL), the product [Pr(hfac)_3_Co(acac)_3_] (0.334 g; 0.30 mmol; 57% yield) was recovered. The elemental analysis calculated for [Pr(hfac)_3_Co(acac)_3_] (C_30_H_24_CoF_18_O_12_Pr) was as follows: C: 32.2% and H: 2.2%; found C: 31.8% and H: 2.2%. IR-ATR (range 1700–650 cm^−1^); 1667 (w), 1650 (s), 1585 (m), 1568 (m), 1558 (m), 1513 (s), 1474 (s), 1430 (w), 1374 (s), 1324 (w), 1279 (w), 1251 (s), 1194 (s), 1138 (s), 1098 (s), 1025 (s), 942 (m), 796 (s), 768 (w), 740 (m), 697 (w), 680 (m) and 658 (s).

The ^1^H NMR (CD_2_Cl_2_, ppm) was as follows: 7.76 (s, H^2^, 9H), 7.55 (s, H^1^, 3H), 5.19 (s, H^3^, 3H), 0.52 (s, H^4^, 9H), (Figure 12).

The ^19^F NMR (CD_2_Cl_2_, ppm) was as follows: −76.71 (s, 18F).

The synthesis of **1Sm** was performed as follows: Starting from [Sm(hfac)_3_] (0.224 g; 0.29 mmol) and [Co(acac)_3_] (0.096 g; 0.27 mmol) in toluene (40 mL), the product [Sm(hfac)_3_Co(acac)_3_] (0.226 g; 0.20 mmol; 74% yield) was recovered. The elemental analysis calculated for [Sm(hfac)_3_Co(acac)_3_] (C_30_H_24_CoF_18_O_12_Sm) was as follows: C: 32.0% and H: 2.1%; found C: 32.1% and H: 1.9%. IR-ATR (range 1700–650 cm^−1^); 1650 (s), 1585 (m), 1567 (m), 1558 (m), 1513 (s), 1475 (m), 1427 (w), 1374 (s), 1324 (w), 1279 (w), 1251 (s), 1194 (s), 1138 (s), 1100 (s), 1027 (m), 943 (m), 795 (s), 766 (w), 740 (m), 697 (w), 678 (m) and 657 (s).

The ^1^H NMR (CD_2_Cl_2_, ppm) was as follows: 6.46 (s, H^1^, 3H), 5.64 (s, H^3^, 3H), 2.69 (s, H^4^, 9H), 2.08 (s, H^2^, 9H), (Figure 12).

The ^19^F NMR (CD_2_Cl_2_, ppm) was as follows: −77.63 (s, 18F).

The synthesis of **1Dy** was performed as follows: Starting from [Dy(hfac)_3_] (0.106 g; 0.14 mmol) and [Co(acac)_3_] (0.048 g; 0.13 mmol) in toluene (40 mL), the product [Dy(hfac)_3_Co(acac)_3_] (0.097 g; 0.09 mmol; 63% yield) was recovered. The elemental analysis calculated for [Dy(hfac)_3_Co(acac)_3_] (C_30_H_24_CoDyF_18_O_12_) was as follows: C: 31.6% and H: 2.1%; found C: 31.8% and H: 2.1%. IR-ATR (range 1700–650 cm^−1^); 1651 (s), 1583 (m), 1561 (m), 1512 (s), 1478 (m), 1428 (w), 1374 (s), 1324 (w), 1276 (w), 1251 (s), 1194 (s), 1136 (s), 1102 (s), 1026 (m), 943 (m), 796 (s), 741 (m), 701 (w), 683 (m) and 657 (s).

The synthesis of [(Ln(hfac)_3_)_2_Co(acac)_3_] (Ln = La, Pr, Sm and Dy) **2Ln** is described below:

The synthesis of the lanthanum derivative [(La(hfac)_3_)_2_Co(acac)_3_] (**2La**) is described at length; the same experimental protocol was used for the other **2Ln** compounds.

The synthesis of **2La** was performed as follows: To a suspension of [La(hfac)_3_] (0.538 g; 0.71 mmol) in DCE (40 mL), [Co(acac)_3_] (0.111 g; 0.31 mmol) was added. After 30′ stirring at 60 °C a green solution was obtained with traces of a colorless solid. After filtration, the volatile phases were completely removed at reduced pressure, obtaining a homogeneous green residue. The product **2La** can be recrystallized from hot toluene (0.327 g; 0.17 mmol 55% yield). The elemental analysis calculated for [(La(hfac)_3_)_2_Co(acac)_3_] (C_45_H_27_CoF_36_La_2_O_18_) was as follows: C: 28.8% and H: 1.4%; found C: 28.5% and H: 1.4%. ATR-IR (range 1700–650 cm^−1^): 1646 (s), 1608 (w), 1558 (w), 1530 (s), 1462 (s), 1345 (m), 1288 (w), 1250 (s), 1196 (s), 1137 (s), 1097 (s), 1029 (m), 949 (m), 940 (m), 800 (s), 769 (w), 739 (m), 697 (w), 684 (w) and 658 (s). This product is not stable when exposed to moisture; it also decomposes in a solid state even after short-term air exposures, as evidenced by gradually changing IR spectra with time when the sample is exposed to air. This compound is highly soluble in chlorinated solvents; the solubility in toluene sensibly increases with temperature.

**Figure 13:**
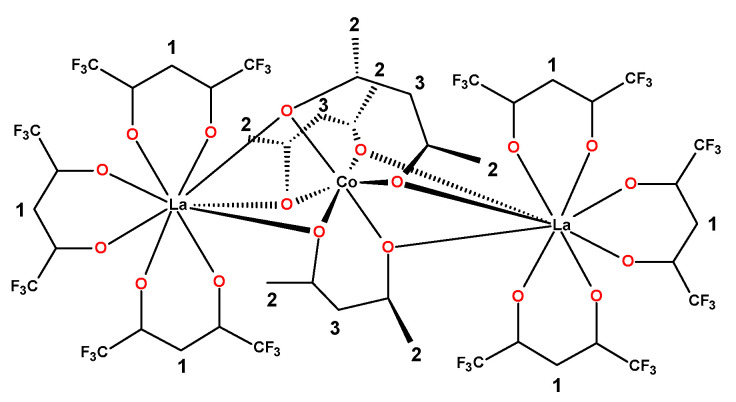
Numbering of ^1^H NMR protons in [(La(hfac)_3_)_2_Co(acac)_3_].

The ^1^H NMR (CDCl_3_, ppm) was as follows: 6.21 (s, H^1^, 6H), 5.97 (s, H^3^, 3H), 2.16 (s, H^2^, 18H), (Figure 13).

The ^19^F NMR (CDCl_3_, ppm) was as follows: −77.11 (s, 36F).

The synthesis of **2Pr** was performed as follows: Starting from [Pr(hfac)_3_] (0.443 g; 0.58 mmol) and [Co(acac)_3_] (0.090 g; 0.25 mmol) in DCE (40 mL), the product [(Pr(hfac)_3_)_2_Co(acac)_3_] (0.319 g; 0.17 mmol; 68% yield) was recovered. The elemental analysis calculated for [(Pr(hfac)_3_)_2_Co(acac)_3_] (C_45_H_27_CoF_36_O_18_Pr_2_) was as follows: C: 28.7% and H: 1.4%; found C: 28.6% and H: 1.3%. IR-ATR (range 1700–650 cm^−1^); 1646 (s), 1605 (w), 1558 (w), 1529 (s), 1460 (s), 1344 (s), 1289 (w), 1248 (s), 1195 (s), 1135 (s), 1098 (s), 1027 (m), 947 (m), 800 (s), 771 (w), 736 (m), 697 (w), 684 (w) and 658 (s). The crystal data (**2Pr**) were as follows: rhombohedral: a = 18.1259(16) Å, b = 18.1259(16) Å, c = 18.456(2) Å, α = 90°, β = 90°, γ = 120° V = 5251.31 Å^3^.

The synthesis of **2Sm** was performed as follows: Starting from [Sm(hfac)_3_] (0.294 g; 0.38 mmol) and [Co(acac)_3_] (0.064 g; 0.18 mmol) in DCE (40 mL), the product [(Sm(hfac)_3_)_2_Co(acac)_3_] (0.132 g; 0.07 mmol; 39% yield) was recovered. The elemental analysis calculated for [(Sm(hfac)_3_)_2_Co(acac)_3_] (C_45_H_27_CoF_36_O_18_Sm_2_) was as follows: C: 28.5% and H: 1.4%; found C: 28.2% and H: 1.4%. IR-ATR (range 1700–650 cm^−1^); 1645 (s), 1608 (w), 1558 (w), 1529 (s), 1462 (s), 1346 (s), 1287 (w), 1247 (s), 1194 (s), 1133 (s), 1096 (m), 1029 (m), 948 (m), 801 (s), 772 (w), 740 (m), 684 (m) and 658 (s). The crystal data (**2Sm**) were as follows: rhombohedral: a = 18.0656(16) Å, b = 18.0656(16) Å, c = 18.3185(8) Å, α = 90°, β = 90°, γ = 120° V = 5177.56 Å^3^.

The synthesis of **2Dy** was performed as follows: Starting from [Dy(hfac)_3_] (0.364 g; 0.47 mmol) and [Co(acac)_3_] (0.081 g; 0.23 mmol) in DCE (40 mL), the product [(Dy(hfac)_3_)_2_Co(acac)_3_] (0.171 g; 0.09 mmol; 39% yield) was recovered. The elemental analysis calculated for [(Dy(hfac)_3_)_2_Co(acac)_3_] (C_45_H_27_CoDy_2_F_36_O_18_) was as follows: C: 28.1% and H: 1.4%; found C: 27.6% and H: 1.7%. IR-ATR (range 1700–650 cm^−1^); 1650 (s), 1559 (w), 1524 (s), 1469 (s), 1368 (m), 1351 (m), 1282 (w), 1249 (s), 1196 (s), 1135 (s), 1099 (m), 1028 (m), 942 (m), 868 (w), 800 (s), 772 (w), 739 (m), 704 (w), 686 (m) and 658 (s).

The synthesis of [La(hfac)_3_Ru(acac)_3_] was performed as follows:

To a suspension of [La(hfac)_3_] (0.351 g; 0.46 mmol) in toluene (25 mL), [Ru(acac)_3_] (0.184 g; 0.46 mmol) was added. After 30′ stirring at 60 °C, a red solution was obtained. After removing the volatiles at reduced pressure, a solid red residue was recovered (0.277 g; 0.24 mmol; 52% yield for [La(hfac)_3_Ru(acac)_3_]. The compound can be recrystallized from a saturated heptane solution. The elemental analysis calculated for [La(hfac)_3_Ru(acac)_3_] (C_30_H_24_F_18_LaO_12_Ru) was as follows: C: 31.1% and H: 2.1%; found C: 31.0% and H: 1.9%. ATR-IR (range 1700–650 cm^−1^): 1650 (s), 1557 (m), 1525 (m), 1503 (m), 1474 (m), 1424 (w), 1370 (m), 1252 (s), 1199 (s), 1138 (s), 1097 (s), 1025 (m), 934 (m), 798 (s), 766 (w), 740 (m), 685 (m) and 658 (m). It is stable for short-term air exposures, as evidenced by the almost over-imposable IR spectra of the sample in air. The product is highly soluble in chlorinated solvents and toluene but scarcely soluble in heptane.

The ^1^H NMR (CD_2_Cl_2_, ppm) was as follows: 7.70 (s, H^1^, 3H), -0.20 (s, H^2^, 9H), -6.49 (s, H^4^, 9H), -28.43 (s, H^3^, 3H), (Figure 12 and Figure S3).

The ^19^F NMR (CD_2_Cl_2_, ppm) was as follows: −76.11 (s, 18F).

The synthesis of [(Ln(hfac)_3_)_2_Ru(acac)_3_] was performed as follows:

To a suspension of [La(hfac)_3_] (0.487 g; 0.64 mmol) in dichloroethane (75 mL), [Ru(acac)_3_] (0.119 g; 0.30 mmol) was added. After 30′ stirring at 60 °C, a red solution was obtained with traces of a colorless solid. After filtration, the volatile phases were completely removed at reduced pressure, obtaining a homogeneous red residue. The product can be recrystallized from hot toluene (0.413 g; 0.22 mmol 72% yield for [(La(hfac)_3_)_2_Ru(acac)_3_]. The elemental analysis calculated for [(La(hfac)_3_)_2_Ru(acac)_3_] (C_45_H_27_F_36_La_2_RuO_18_) was as follows: C: 28.2% and H: 1.4%; found C: 28.5% and H: 1.2%. ATR-IR (range 1700–650 cm^−1^): 1645 (s), 1610 (w), 1557 (w), 1528 (m), 1461 (s), 1345 (m), 1249 (s), 1196 (s), 1137 (s), 1096 (s), 1025 (m), 935 (m), 802 (s), 772 (w), 739 (m), 690 (w), 671 (w) and 657 (s). This product is not stable when exposed to moisture; it also decomposes in a solid state even after short-term air exposures, as evidenced by gradually changing IR spectra with time when the sample is exposed to air. The product, soluble in chlorinated solvents and in hot toluene, is highly air-sensitive in solution. Hydrolysis occurs even in the solid state as evidenced by IR changes after short-term air exposures.

The computational details are described below:

DFT calculations were carried out by using the Orca suite of programs (version 5.0.0) [55]. The complexes were optimized using GGA PBE functional [56]; Coulomb and exchange integrals in hybrid calculations were approximated by using the Resolution of Identity approximation with the def2/JK auxiliary basis set [57]. Dispersion corrections were included by adopting Grimme’s DFT-D3 method [58]. Each lanthanide was considered in its +3 oxidation state; La(III), Pr(III) and Sm(III), had 0, 2 and 5 unpaired electrons, while Co(III) and Ru(III) had zero and one unpaired electron, respectively. All attempts to obtain stable optimized heterodinuclear or heterotrinuclear structures with Dy as lanthanide failed.

## 4. Conclusions

A mononuclear octahedral symmetric *tris*-chelate [M(acac)_3_] complex can behave as a tridentate capping ligand, facially coordinated to a formally unsaturated [Ln(hfac)_3_] and afford heterodinuclear d/f complexes. The major achievement of this work establishes that it is possible for an octahedral [M(acac)_3_] complex (M = Co or Ru), featuring two equivalent opposite triangular faces of donor oxygen atoms, to present donor properties towards two unsaturated lanthanide fragments. Heterotrinuclear sandwich d/f compounds [(Ln(hfac)_3_)_2_M(acac)_3_] can be obtained by exploiting a Lewis acid-to-Lewis base interaction in strictly anhydrous conditions. The Co heterotrinuclear complexes show a progressive decrease in stability across the lanthanide series, which is evidenced by both experimental and computational data. Indeed, quantum mechanical calculations make it possible, on the one hand, to support the hypothesis of the structural arrangement obtained from spectroscopic data and, on the other hand, to rationalize the high susceptibility of the heterotrinuclear sandwich complexes to hydrolysis. The synthetic protocol is potentially applicable across the lanthanide series using [Co(acac)_3_], and it is also applicable to the synthesis of complexes with a different Lewis base [Ru(acac)_3_]. The comparison between the lanthanum sandwich complexes of Co and Ru reveals that the latter exhibits a lower stability. This conclusion is supported by the calculated ∆G values, with the Co value being greater than that of Ru by 3.5 kcal/mol. Although a marked reactivity of these complexes to moisture is observed even in the solid state, these results suggest that a new family of molecular heteronuclear d/f compounds with modulable photophysical or magnetic properties can be potentially prepared under anhydrous conditions.

## Figures and Tables

**Table 1:** Geometric parameters (Ln‧Co and bond lengths in Å) for [Ln(hfac)_3_Co(acac)_3_] and [(Ln(hfac)_3_)_2_Co(acac)_3_] complexes (Ln = La, Pr, Sm). The Mayer bond order (MBO) index is also reported.

	Distance	Bond Lengths		Mayer Bond Order (MBO)	
	Ln⋯Co	Ln-O_bridge_	Co-O_bridge_	Ln-O_bridge_	Co-O_bridge_
[Co(acac)_3_]	//	//	1.901	//	0.665
[La(hfac)_3_Co(acac)_3_]	3.419	2.701	1.914	0.160	0.530
[Pr(hfac)_3_Co(acac)_3_]	3.383	2.670	1.911	0.190	0.551
[Sm(hfac)_3_Co(acac)_3_]	3.367	2.654	1.908	0.188	0.557
[(La(hfac)_3_)_2_Co(acac)_3_]	3.471	2.759	1.904	0.126	0.587
[(Pr(hfac)_3_)_2_Co(acac)_3_]	3.437	2.732	1.901	0.170	0.597
[(Sm(hfac)_3_)_2_Co(acac)_3_]	3.427	2.720	1.900	0.173	0.604

**Table 2:** Calculated ∆E and ∆G values (kcal/mol) for the [Ln(hfac)_3_Co(acac)_3_] and [(Ln(hfac)_3_)_2_Co(acac)_3_] complexes (Ln = La, Pr, Sm) at 298.15 K.

Product	Reagents	∆E	∆G
[La(hfac)_3_Co(acac)_3_]	[Co(acac)_3_] and [La(hfac)_3_]	−45.2	−28.4
[Pr(hfac)_3_Co(acac)_3_]	[Co(acac)_3_] and [Pr(hfac)_3_]	−39.2	−22.5
[Sm(hfac)_3_Co(acac)_3_]	[Co(acac)_3_] and [Sm(hfac)_3_]	−37.5	−20.8
[(La(hfac)_3_)_2_Co(acac)_3_]	[Co(acac)_3_] and 2[La(hfac)_3_]	−81.7	−47.0
[(Pr(hfac)_3_)_2_Co(acac)_3_]	[Co(acac)_3_] and 2[Pr(hfac)_3_]	−71.5	−36.8
[(Sm(hfac)_3_)_2_Co(acac)_3_]	[Co(acac)_3_] and 2[Sm(hfac)_3_]	−65.6	−30.9
[(La(hfac)_3_)_2_Co(acac)_3_]	[La(hfac)_3_Co(acac)_3_] and [La(hfac)_3_]	−36.5	−18.6
[(Pr(hfac)_3_)_2_Co(acac)_3_]	[Pr(hfac)_3_Co(acac)_3_] and [Pr(hfac)_3_]	−32.3	−14.3
[(Sm(hfac)_3_)_2_Co(acac)_3_]	[Sm(hfac)_3_Co(acac)_3_] and [Sm(hfac)_3_]	−28.1	−10.1

## Data Availability

Data is contained within the article or Supplementary Material.

## Supplementary Materials

The following supporting information can be downloaded at https://www.mdpi.com/article/10.3390/molecules29163927/s1, Figure S1. The ^1^H (left) and ^19^F (right) NMR spectrum of [Pr(hfac)_3_Co(acac)_3_], **1Pr**, in CD_2_Cl_2._ Figure S2. The ^1^H (left) and ^19^F (right) NMR spectrum of [Sm(hfac)_3_Co(acac)_3_], **1Sm**, in CD_2_Cl_2_. Figure S3. The ^1^H (left) and ^19^F (right) NMR spectrum of [La(hfac)_3_Ru(acac)_3_] in CD_2_Cl_2._ Figure S4. The ^1^H and ^19^F NMR spectra of [(Sm(hfac)_3_)_2_Co(acac)_3_], **2Sm**, in CD_2_Cl_2_: (i) immediately after preparation (black) and (ii) after 30′ (brown). Figure S5. The ^1^H NMR spectrum of [Sm(hfac)_3_(H_2_O) _2_] in CD_2_Cl_2_. X-ray diffraction studies. Table S1. Crystal data and refinement summaries for **1Sm**. Figure S6. Molecular structure of [Sm(hfac)_3_Co(acac)_3_], **1Sm**. Figure S7. Polyhedral representation of coordination centers of **1Sm**. Table S2. Selected bond angles (°) in **1Sm**. Table S3. Selected bond lengths (Å) in **1Sm**. Figure S8. Void representation in the unit cell along the a-axis of **1Sm**. Figure S9. Optimized structures of [Ln(hfac)_3_Co(acac)_3_] with Ln = La, Pr and Sm. The green, grey, red and violet spheres are F, C, O and Co atoms, respectively. La is cyan, Pr is yellow and Sm is pink. H atoms are omitted for clarity. Figure S10. Optimized structures of [(Ln(hfac)_3_)_2_Co(acac)_3_] with Ln = La, Pr and Sm. The green, grey, red and violet spheres are F, C, O and Co atoms, respectively. La is cyan, Pr is yellow and Sm is pink. H atoms are omitted for clarity. Figure S11. Optimized structures of [La(hfac)_3_Ru(acac)_3_] and [(La(hfac)_3_)_2_Ru(acac)_3_] The cyan, green, grey red and dark green-violet spheres are La, F, C, O and Ru atoms, respectively. H atoms are omitted for clarity. Table S4: Bond lengths in Å and MBO for La-O_bridge_ and M-O_bridge_ in the [La(hfac)_3_M(acac)_3_] and [(Ln(hfac)_3_)_2_M(acac)_3_] complexes (M = Co, Ru). Table S5: Calculated ∆E and ∆G values (kcal/mol) for the [La(hfac)_3_M(acac)_3_] and [(La(hfac)_3_)_2_M(acac)_3_] complexes (M = Co, Ru). Table S6: Assignment of the observed IR band to vibrational modes for [La(hfac)_3_Co(acac)_3_]. Table S7: Assignment of the observed IR band to vibrational modes for [(La(hfac)_3_)_2_Co(acac)_3_]. Figure S12: Comparison of the calculated and experimental spectra for [La(hfac)_3_Co(acac)_3_] and [(La(hfac)_3_)_2_Co(acac)_3_]. Figure S13: Comparison of the calculated and experimental spectra for [La(hfac)_3_Ru(acac)_3_] and [(La(hfac)_3_)_2_Ru(acac)_3_]. References [59,60,61,62,63,64,65] are cited in Supplementary Materials.
