# Supplementary material for: Sandwich d/f Heterometallic Complexes [(Ln(hfac)3)2M(acac)3] (Ln = La, Pr, Sm, Dy and M = Co; Ln = La and M = Ru)
Source: Molecules. 2024 Aug 20;29(16):3927. doi: 10.3390/molecules29163927 (PMC11356967; doi:10.3390/molecules29163927)

checkCIF/PLATON report

Structure factors have been supplied for datablock(s) mo_COAT178_0m_a

THIS REPORT IS FOR GUIDANCE ONLY. IF USED AS PART OF A REVIEW PROCEDURE FOR PUBLICATION, IT SHOULD NOT REPLACE THE EXPERTISE OF AN EXPERIENCED CRYSTALLOGRAPHIC REFEREE.

No syntax errors found. CIF dictionary Interpreting this report

Datablock: mo_COAT178_0m_a

Bond precision:	C-C = 0.0092 A		Wavelength=0.71073
Cell:	a=18.7578(7)	b=22.5503(9)	c=21.2002(9)
	alpha=90	beta=110.967(1)	gamma=90
Temperature:	100 K		

	Calculated	Reported
Volume	8373.8(6)	8373.8(6)
Space group	P 21/c	P 21/c
Hall group	-P 2ybc	-P 2ybc
Moiety formula	C30 H24 Co F18 O12 Sm	C30 H24 Co F18 O12 Sm
Sum formula	C30 H24 Co F18 O12 Sm	C30 H24 Co F18 O12 Sm
Mr	1127.78	1127.77
Dx,g cm-3	1.789	1.789
Z	8	8
Mu (mm-1)	1.919	1.919
F000	4408.0	4408.0
F000'	4413.27	
h,k,lmax	22,26,25	22,26,25
Nref	14808	14796
Tmin,Tmax	0.853,0.912	0.667,0.745
Tmin'	0.853	

Correction method= # Reported T Limits: Tmin=0.667 Tmax=0.745
AbsCorr = MULTI-SCAN

Data completeness= 0.999 Theta(max)= 25.037

R(reflections)= 0.0436(11166) wR2(reflections)= 0.1014(14796)

S = 1.014 Npar= 1115

The following ALERTS were generated. Each ALERT has the format
test-name_ALERT_alert-type_alert-level.
Click on the hyperlinks for more details of the test.

Alert level C

```
PLAT213_ALERT_2_C Atom F6B             has ADP max/min Ratio .....         3.4 oblate
PLAT220_ALERT_2_C NonSolvent   Resd 2  F   Ueq(max)/Ueq(min) Range         4.0 Ratio
PLAT230_ALERT_2_C Hirshfeld Test Diff for    F36      --C49      .         5.2 s.u.
PLAT234_ALERT_4_C Large Hirshfeld Difference F6A      --C53      .        0.16 Ang.
PLAT342_ALERT_3_C Low Bond Precision on  C-C Bonds ..............     0.00921 Ang.
PLAT601_ALERT_2_C Unit Cell Contains Solvent Accessible VOIDS of .          43 Ang**3
PLAT910_ALERT_3_C Missing # of FCF Reflection(s) Below Theta(Min).          5 Note
                1  0  0,   1  1  0,   0  2  0,  -1  1  1,   0  1  1,
PLAT911_ALERT_3_C Missing FCF Refl Between Thmin & STh/L=    0.595          6 Report
                2  0  0,  13 21  0,   9 24  1,  -8 23 12, -20  9 15, -15 12 20,
```

Alert level G

```
PLAT083_ALERT_2_G SHELXL Second Parameter in WGHT  Unusually Large       40.99 Why ?
PLAT171_ALERT_4_G The CIF-Embedded .res File Contains EADP Records           2 Report
PLAT177_ALERT_4_G The CIF-Embedded .res File Contains DELU Records           1 Report
PLAT230_ALERT_2_G Hirshfeld Test Diff for    F9A      --C43      .         6.0 s.u.
PLAT230_ALERT_2_G Hirshfeld Test Diff for    F7B      --C53      .         8.2 s.u.
PLAT230_ALERT_2_G Hirshfeld Test Diff for    F12B     --C43      .         8.2 s.u.
PLAT230_ALERT_2_G Hirshfeld Test Diff for    F2A      --C17      .         7.3 s.u.
PLAT230_ALERT_2_G Hirshfeld Test Diff for    F14A     --C15      .         8.0 s.u.
PLAT230_ALERT_2_G Hirshfeld Test Diff for    F21A     --C15      .        10.2 s.u.
PLAT230_ALERT_2_G Hirshfeld Test Diff for    F23A     --C19      .         8.6 s.u.
PLAT230_ALERT_2_G Hirshfeld Test Diff for    F2B      --C17      .         7.6 s.u.
PLAT230_ALERT_2_G Hirshfeld Test Diff for    F14B     --C15      .         6.8 s.u.
PLAT230_ALERT_2_G Hirshfeld Test Diff for    F15B     --C17      .         6.6 s.u.
PLAT230_ALERT_2_G Hirshfeld Test Diff for    F21B     --C15      .         5.6 s.u.
PLAT230_ALERT_2_G Hirshfeld Test Diff for    F23B     --C19      .         5.3 s.u.
PLAT242_ALERT_2_G Low    'MainMol' Ueq as Compared to Neighbors of         C45 Check
PLAT242_ALERT_2_G Low    'MainMol' Ueq as Compared to Neighbors of         C47 Check
PLAT242_ALERT_2_G Low    'MainMol' Ueq as Compared to Neighbors of         C49 Check
PLAT242_ALERT_2_G Low    'MainMol' Ueq as Compared to Neighbors of         C51 Check
PLAT242_ALERT_2_G Low    'MainMol' Ueq as Compared to Neighbors of         C53 Check
PLAT242_ALERT_2_G Low    'MainMol' Ueq as Compared to Neighbors of         C13 Check
PLAT242_ALERT_2_G Low    'MainMol' Ueq as Compared to Neighbors of         C15 Check
PLAT242_ALERT_2_G Low    'MainMol' Ueq as Compared to Neighbors of         C17 Check
PLAT242_ALERT_2_G Low    'MainMol' Ueq as Compared to Neighbors of         C19 Check
PLAT242_ALERT_2_G Low    'MainMol' Ueq as Compared to Neighbors of         C21 Check
PLAT242_ALERT_2_G Low    'MainMol' Ueq as Compared to Neighbors of         C23 Check
PLAT301_ALERT_3_G Main Residue  Disorder ..............(Resd    1)         10% Note
PLAT301_ALERT_3_G Main Residue  Disorder ..............(Resd    2)          8% Note
PLAT367_ALERT_2_G Long?  C(sp?)-C(sp?) Bond  C44       - C45     .        1.54 Ang.
PLAT434_ALERT_2_G Short Inter HL..HL Contact F16      ..F7B      .        2.82 Ang.
                                            1-x,1-y,1-z  =      3_666 Check
PLAT794_ALERT_5_G Tentative Bond Valency for Sm1       (III)     .        3.46 Info
PLAT794_ALERT_5_G Tentative Bond Valency for Sm2       (III)     .        3.48 Info
PLAT794_ALERT_5_G Tentative Bond Valency for Co1       (III)     .        3.14 Info
PLAT794_ALERT_5_G Tentative Bond Valency for Co2       (III)     .        3.15 Info
PLAT909_ALERT_3_G Percentage of I>2sig(I) Data at Theta(Max) Still         58% Note
PLAT969_ALERT_5_G The 'Henn et al.' R-Factor-gap value ..........       2.151 Note
```

```
              Predicted wR2: Based on SigI**2  4.71 or SHELX Weight 10.00
PLAT978_ALERT_2_G Number C-C Bonds with Positive Residual Density.          0 Info
```

```
  0 ALERT level A = Most likely a serious problem - resolve or explain
  0 ALERT level B = A potentially serious problem, consider carefully
  8 ALERT level C = Check. Ensure it is not caused by an omission or oversight
 37 ALERT level G = General information/check it is not something unexpected

  0 ALERT type 1 CIF construction/syntax error, inconsistent or missing data
 31 ALERT type 2 Indicator that the structure model may be wrong or deficient
  6 ALERT type 3 Indicator that the structure quality may be low
  3 ALERT type 4 Improvement, methodology, query or suggestion
  5 ALERT type 5 Informative message, check
```

It is advisable to attempt to resolve as many as possible of the alerts in all categories. Often the minor alerts point to easily fixed oversights, errors and omissions in your CIF or refinement strategy, so attention to these fine details can be worthwhile. In order to resolve some of the more serious problems it may be necessary to carry out additional measurements or structure refinements. However, the purpose of your study may justify the reported deviations and the more serious of these should normally be commented upon in the discussion or experimental section of a paper or in the "special_details" fields of the CIF. checkCIF was carefully designed to identify outliers and unusual parameters, but every test has its limitations and alerts that are not important in a particular case may appear. Conversely, the absence of alerts does not guarantee there are no aspects of the results needing attention. It is up to the individual to critically assess their own results and, if necessary, seek expert advice.

Publication of your CIF in IUCr journals

A basic structural check has been run on your CIF. These basic checks will be run on all CIFs submitted for publication in IUCr journals (*Acta Crystallographica*, *Journal of Applied Crystallography*, *Journal of Synchrotron Radiation*); however, if you intend to submit to *Acta Crystallographica Section C* or *E* or *IUCrData*, you should make sure that full publication checks are run on the final version of your CIF prior to submission.

Publication of your CIF in other journals

Please refer to the *Notes for Authors* of the relevant journal for any special instructions relating to CIF submission.

PLATON version of 15/07/2024; check.def file version of 15/07/2024

Datablock mo_COAT178_0m_a - ellipsoid plot

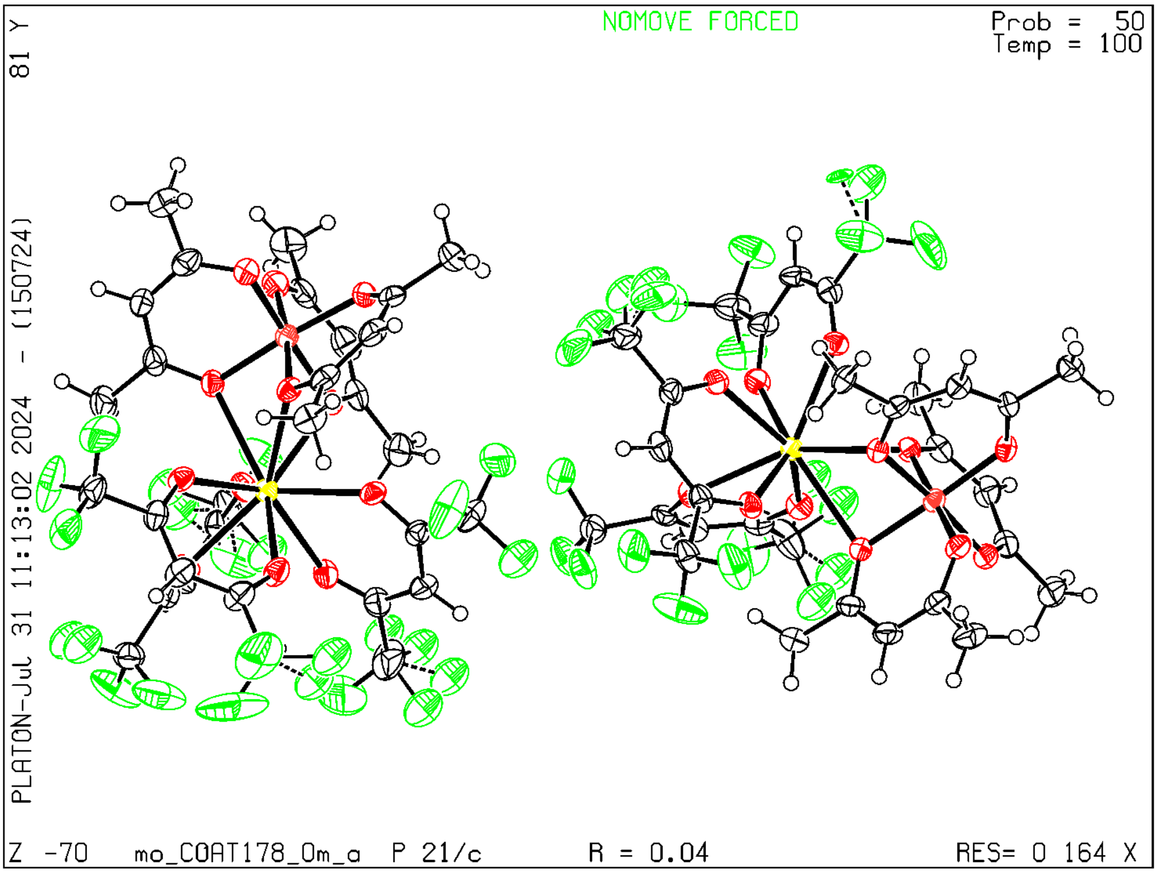

Supplement: Supplementary file 1 [file molecules-29-03927-s001.zip › checkcifcoat178.pdf]
