# Supplementary material for: Sandwich d/f Heterometallic Complexes [(Ln(hfac)3)2M(acac)3] (Ln = La, Pr, Sm, Dy and M = Co; Ln = La and M = Ru)
Source: Molecules. 2024 Aug 20;29(16):3927. doi: 10.3390/molecules29163927 (PMC11356967; doi:10.3390/molecules29163927)
Supplement: Supplementary file 1 [file molecules-29-03927-s001.zip › molecules-3107254-supplementary.pdf]

Molecules

Sandwich d/f Heterometallic Complexes [(Ln(hfac)$_3$)$_2$M(acac)$_3$] (Ln = La, Pr, Sm, Dy and M = Co; Ln = La and M = Ru)

Cristian Grechi [1], Silvia Carlotto [2,3], Massimo Guelfi [1,4], Simona Samaritani [1,4], Lidia Armelao [3,5] and Luca Labella [1,4,*]

1 Dipartimento di Chimica e Chimica Industriale and CIRCC, Università di Pisa, via Giuseppe Moruzzi 13, 56124 Pisa, Italy; cristian.grechi@libero.it (C.G.); massimo.guelfi@unipi.it (M.G.); simona.samaritani@unipi.it (S.S.)
2 Istituto di Chimica della Materia Condensata e di Tecnologie per l'Energia (ICMATE), Consiglio Nazionale delle Ricerche (CNR) e INSTM, Presso Dipartimento di Scienze Chimiche, Università di Padova, via Marzolo 1, 35131 Padova, Italy; silvia.carlotto@unipd.it
3 Dipartimento di Scienze Chimiche e INSTM, Università di Padova, via Marzolo 1, 35131 Padova, Italy; lidia.armelao@unipd.it
4 Centro per l'Integrazione della Strumentazione Scientifica dell'Università di Pisa (C.I.S.U.P.), Università di Pisa, 56126 Pisa, Italy
5 Dipartimento di Scienze Chimiche e Tecnologie dei Materiali (DSCTM), Consiglio Nazionale delle Ricerche (CNR), Piazzale A. Moro 7, 00185 Roma, Italy
* Correspondence: luca.labella@unipi.it

Supplementary Information

Table of contents

Figure S1. ^{1}H (left) and ^{19}F (right) NMR spectrum of [Pr(hfac)$_3$Co(acac)$_3$], **1Pr**, in CD_2Cl_2

Figure S2. ^{1}H (left) and ^{19}F (right) NMR spectrum of [Sm(hfac)$_3$Co(acac)$_3$], **1Sm,** in CD_2Cl_2

Figure S3. ^{1}H (left) and ^{19}F (right) NMR spectrum of [La(hfac)$_3$Ru(acac)$_3$] in CD_2Cl_2

Figure S4. ^{1}H and ^{19}F NMR spectra of [(Sm(hfac)$_3$)$_2$Co(acac)$_3$], **2Sm**, in CD_2Cl_2: i) immediately after preparation (black) and ii) after 30' (brown).

Figure S5. ^{1}H NMR spectrum of [Sm(hfac)$_3$(H_2O)$_2$] in CD_2Cl_2

Single-Crystal X-Ray Diffraction studies

Table S1. Crystal data and measurement details for **1Sm**.

Figure S6. Molecular structure of **1Sm.** Displacement parameters are drawn at 30% probability level.

Figure S7. Polyhedral representation of coordination centers of **1Sm.** The fluorine atoms have been omitted for clarity.

Table S2. Selected bond angles (°) in **1Sm**.

Table S3. Selected bond lengths (Å) in **1Sm.**

Figure S8. Voids representation in the unit cell along the a-axis of **1Sm.**

Figure S9. Optimized structures of [Ln(hfac)$_3$Co(acac)$_3$] with Ln=La, Pr and Sm. Green, grey, red and violet spheres are F, C, O and Co atoms, respectively. La is cyan, Pr is yellow and Sm is pink. H atoms are omitted for clarity.

Figure S10. Optimized structures of [(Ln(hfac)$_3$)$_2$Co(acac)$_3$] with Ln=La, Pr and Sm. Green, grey, red and violet spheres are F, C, O and Co atoms, respectively. La is cyan, Pr is yellow and Sm is pink. H atoms are omitted for clarity.

Figure S11. Optimized structures of [La(hfac)$_3$Ru(acac)$_3$] and [(La(hfac)$_3$)$_2$Ru(acac)$_3$] Cyan, green, grey red and dark green violet spheres are La, F, C, O and Ru atoms, respectively. H atoms are omitted for clarity.

Table S4: Bond lengths in Å and MBO for La-O$_{bridge}$ and M-O$_{bridge}$ in [La(hfac)$_3$M(acac)$_3$] and [(Ln(hfac)$_3$)$_2$M(acac)$_3$] complexes (M = Co, Ru).

Table S5: Calculated ΔE and ΔG values (kcal/mol) for [La(hfac)$_3$M(acac)$_3$] and [(La(hfac)$_3$)$_2$M(acac)$_3$] complexes (M= Co, Ru).

Table S6: Assignment of the observed IR band to vibrational modes for [La(hfac)$_3$Co(acac)$_3$]

Table S7: Assignment of the observed IR band to vibrational modes for [(La(hfac)$_3$)$_2$Co(acac)$_3$]

Figure S12: Comparison of calculated and experimental spectra for [La(hfac)$_3$Co(acac)$_3$] (green) and [(La(hfac)$_3$)$_2$Co(acac)$_3$] (black). The three selected colored areas help the reader to better compare the most relevant parts of the IR spectra.

Figure S13: Comparison of calculated and experimental spectra for [La(hfac)$_3$Ru(acac)$_3$] (black) and [(La(hfac)$_3$)$_2$Ru(acac)$_3$] (blue). The three selected colored areas help the reader to better compare the most relevant parts of the IR spectra..

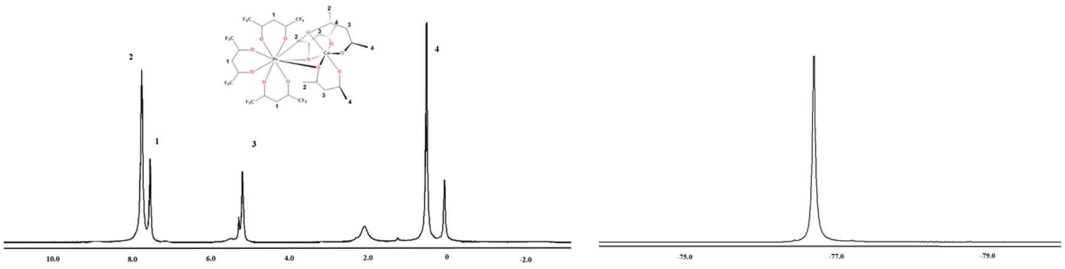

Figure S1. ^{1}H (left) and ^{19}F (right) NMR spectrum of [Pr(hfac)$_3$Co(acac)$_3$], **1Pr**, in CD_2Cl_2

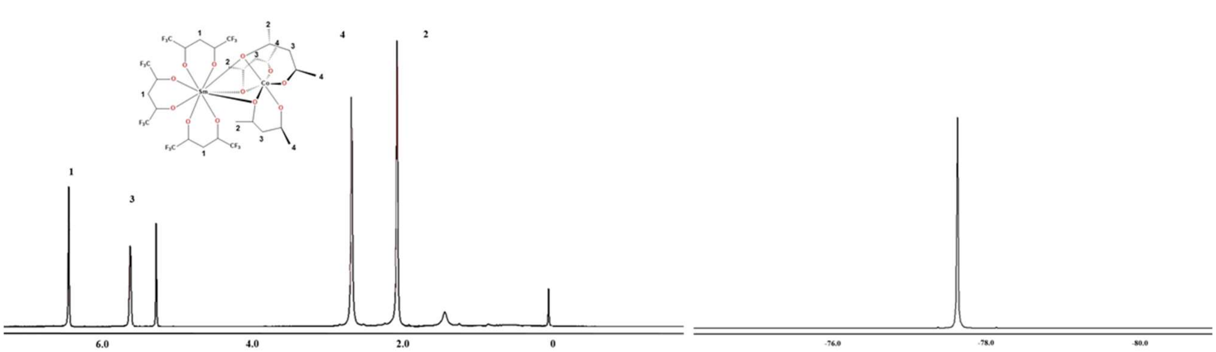

Figure S2. ^{1}H (left) and ^{19}F (right) NMR spectrum of [Sm(hfac)$_3$Co(acac)$_3$], **1Sm,** in CD_2Cl_2

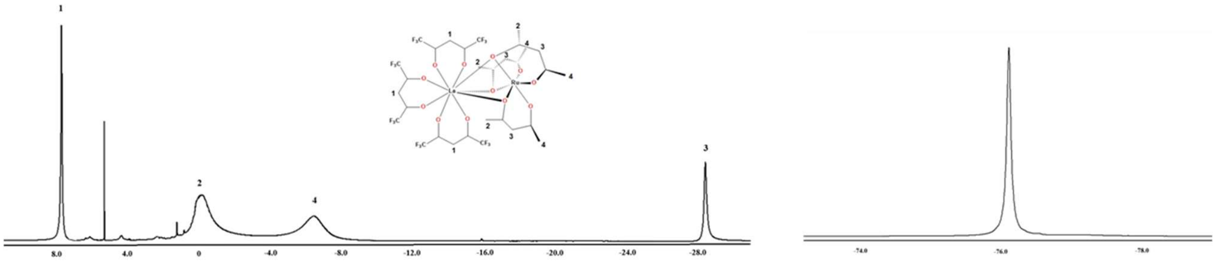

Figure S3. ^{1}H (left) and ^{19}F (right) NMR spectrum of [La(hfac)$_3$Ru(acac)$_3$] in CD_2Cl_2

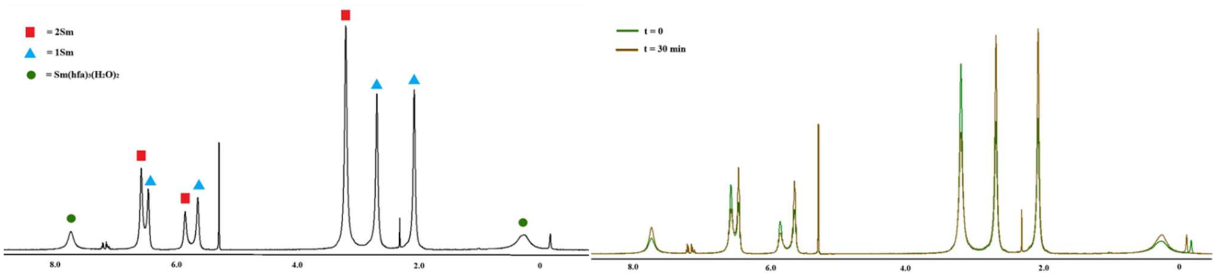

Figure S4. ^{1}H and ^{19}F NMR spectra of [$(Sm(hfac)_3)_2Co(acac)_3$], **2Sm**, in CD_2Cl_2: i) immediately after preparation (green) and ii) after 30′ (brown).

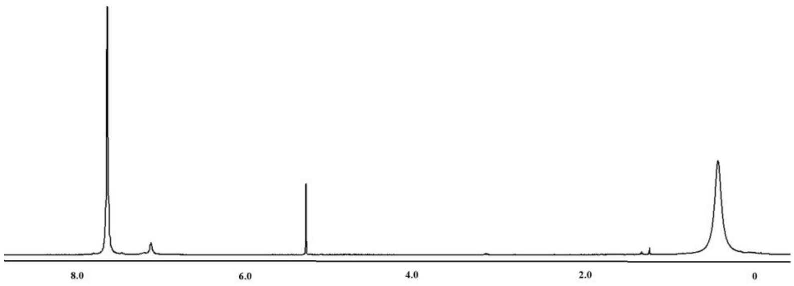

Figure S5. ^{1}H NMR spectrum of [$Sm(hfac)_3(H_2O)_2$] in CD_2Cl_2

Single-Crystal X-Ray Diffraction studies

Single-crystal X-ray diffraction was performed with a Bruker D8 Venture instrument equipped with microfocus Mo source (Kα radiation, λ = 0.71073 Å) and a 2D Photon III detector. The main experimental details regarding the determination of the structure of **1Sm** by single-crystal X-ray diffraction are reported in **Table S1**. The unit cell was identified and initially refined using APEX4.[1]

Successively, data were integrated and reduced using SAINT[2] and XPREP.[3] Absorption effects were corrected using SADABS.[4] Structures were solved and refined with the aid of SHELXL-2019/1.[5] Hydrogen atoms were fixed at calculated positions and refined by using a riding model. The complex **1Sm** crystallizes in a monoclinic space group $P2_1/c$, with Z=8 for the formula unit $C_{30}H_{24}CoF_{18}O_{16}Sm$. The asymmetric unit is composed of two independent dinuclear complexes as shown in **Figure S6**. In each of these, cobalt adopts an octahedral geometry while samarium adopts a tricapped trigonal prism geometry sharing a triangular face defined by three oxygen atoms of three different acetylacetonato ligands (**Figure S7**). The Co(III) center has a *pseudo* octahedral geometry and all bonds angles are in the range between 84.6(2)° and 96.1(2)°. For the samarium(III) center three types of angles can be distinguished: i) those involving only oxygen atoms shared with the cobalt center have angles close to 60°; ii) those involving oxygen atoms belonging to the same hexafluoroacetylacetonato unit have angles close to 70° as well as iii) those between oxygen atoms belonging to both an acetylacetonate and a hexafluoroacetylacetonate (**Table S2**). All Sm-O bond distances are in the range between 2.398(3) Å and 2.577(4) Å while Co-O bond distances vary between 1.869(5) Å and 1.886(5) Å in line with what is reporteed for a similar complex [6] (**Table S3**). The residual charge density (1.484 e/$Å^3$) cannot be attributed to any residual solvent in the crystalline cell since the calculated voids have a total volume of 118.84 A^3 representing 1.4% of the total volume of the cell and in fact, these charges are in proximity to fluorine atoms that cannot be perfectly modeled due to the high disorder in the CF_3 region

(**Figure S8**). Crystallographic data for **1Sm** has been deposited with the Cambridge Crystallographic Data Center as supplementary publication nº. CCDC 2374735 This data can be obtained free of charge from the Cambridge Crystallographic Data Center via www.ccdc.cam.ac.uk/data_request/cif.

Table S1. Crystal data and measurement details for **1Sm**.

Compound	**Sm1**
CCDC ID	2374735
Formula	$C_{30}H_{24}CoF_{18}O_{12}Sm$
FW, g mol^{-1}	1127.77
T, K	100(2)
λ, Å	0.71073
Crystal system	monoclinic
Space group	$P2_1/c$
a, Å	18.7578(7)
b, Å	22.5503(9)
c, Å	21.2002(9)
β,°	110.9670(10)
Cell Volume, Å^3	8373.8(6)
Z	8
D_c, g cm^{-3}	1.789
μ, mm^{-1}	1.919
F(000)	4408
Crystal size, mm	0.083x0.080x0.048
θ limits,°	1.97 to 25.04
Reflections collected	142587
Independent reflections	14796 [R_{int} = 0.1157]
Data / restraints /parameters	14796 / 0 / 1121
Goodness on fit on F^2	1.016
R_1 ($I > 2\sigma(I)$)	0.0434 (11166 data)
wR_2 ($I > 2\sigma(I)$)	0.0893 (11166 data)
R_1 (*all data*)	0.0679
wR_2 (all data)	0.1012
Largest diff. peak and hole, e Å^{-3}	1.484 / -1.600

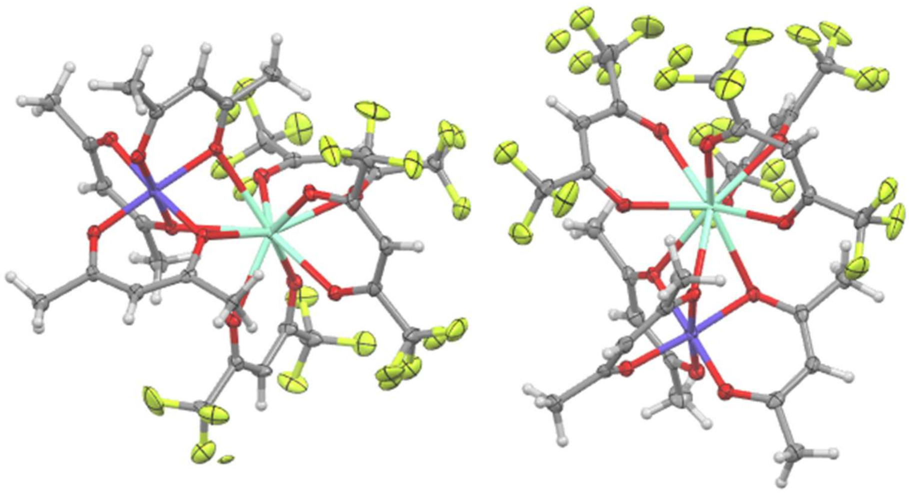

Figure S6. Molecular structure of **1Sm.** Displacement parameters are drawn at 30% probability level.

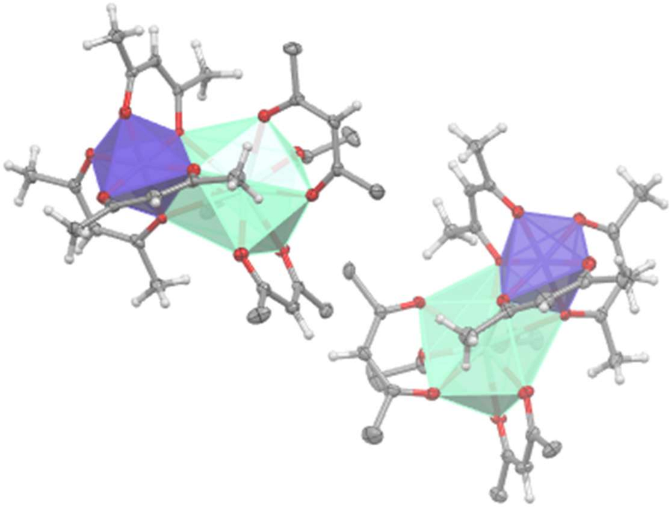

Figure S7. Polyhedral representation of coordination centers of **Sm1.** The fluorine atoms have been omitted for clarity

Table S2. Selected bond angles (°) in **1Sm**.

O1-Sm1-O3	70.4(1)	O5-Co1-O6	96.2(2)
O1-Sm1-O5	70.7(1)	O5-Co1-O7	84.9(2)
O1-Sm1-O12	70.2(1)	O6-Co1-O7	91.2(2)

O2-Sm1-O5	72.6(1)	O6-Co1-O11	86.8(2)
O2-Sm1-O7	71.7(1)	O11-Co1-O12	90.9(2)
O2-Sm1-O14	70.9(1)	O11-Co1-O13	89.0(2)
O5-Sm1-O7	59.7(1)		
O5-Sm1-O12	60.7(1)		
O7-Sm1-O12	59.3(1)		

Table S3. Selected bond lengths (Å) in **1Sm.**

Sm1-O1	2.398(3) 1	Co1-O5	1.886(5) 1
Sm1-O2	2.390(4) 1	Co1-O6	1.865(3) 1
Sm1-O3	2.402(5) 1	Co1-O7	1.890(3) 1
Sm1-O5	2.541(4) 1	Co1-O11	1.869(5) 1
Sm1-O7	2.577(4) 1	Co1-O12	1.881(3) 1
Sm1-O12	2.550(4) 1	Co1-O13	1.871(3) 1
Sm1-O14	2.387(4) 1		
Sm1-O15	2.385(3) 1		
Sm1-O16	2.412(5) 1		

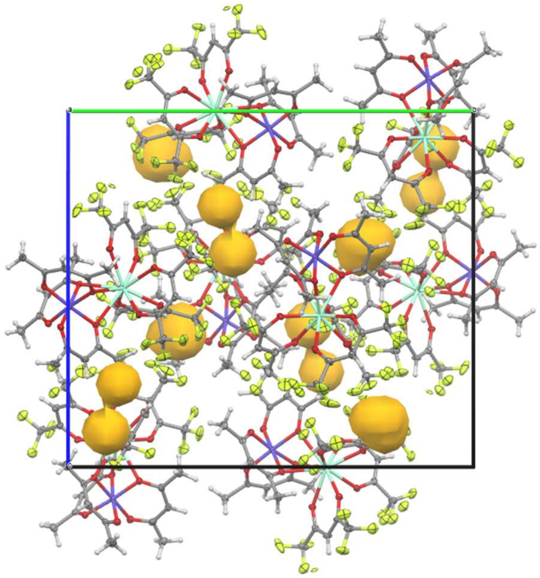

Figure S8. Voids representation in the unit cell along the a-axis of **1Sm.**

Figure S9. Optimized structures of [Ln(hfac)$_3$Co(acac)$_3$] with Ln=La, Pr and Sm. Green, grey, red and violet spheres are F, C, O and Co atoms, respectively. La is cyan, Pr is yellow and Sm is pink. H atoms are omitted for clarity.

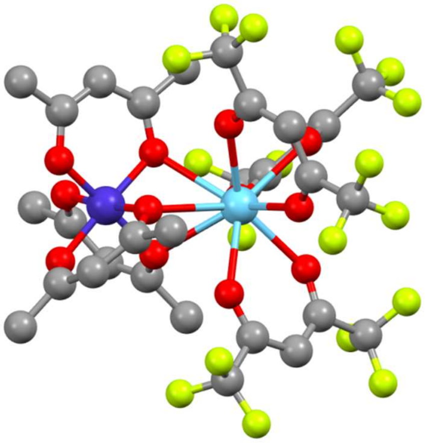

[La(hfac)$_3$Co(acac)$_3$] (1La)

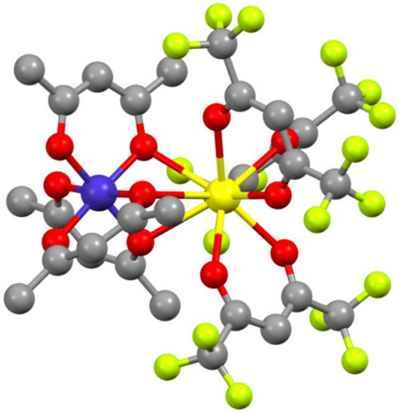

[Pr(hfac)$_3$Co(acac)$_3$] (1Pr)

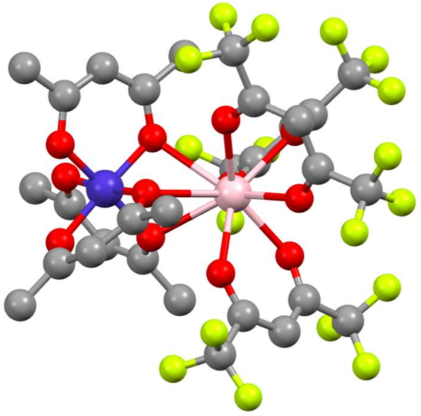

[Sm(hfac)$_3$Co(acac)$_3$] (1Sm)

Figure S10. Optimized structures of [(Ln(hfac)$_3$)$_2$Co(acac)$_3$] with Ln=La, Pr and Sm. Green, grey, red and violet spheres are F, C, O and Co atoms, respectively. La is cyan, Pr is yellow and Sm is pink. H atoms are omitted for clarity.

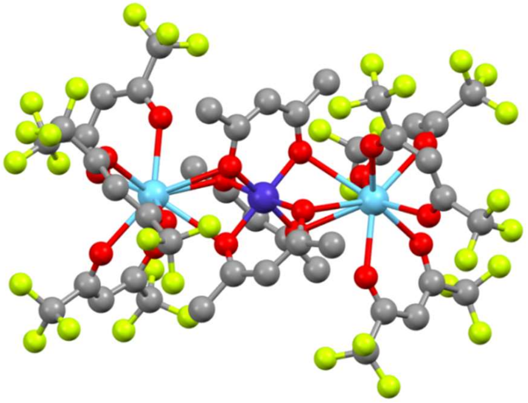

[(La(hfac)$_3$)$_2$Co(acac)$_3$] (2La)

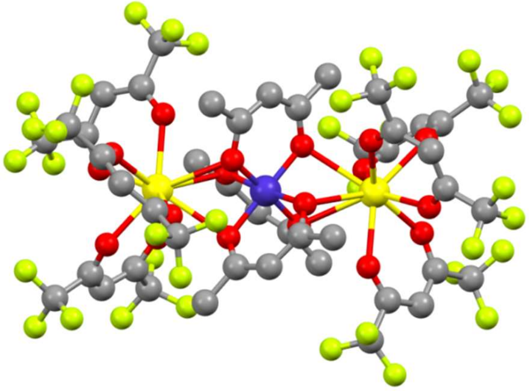

[(Pr(hfac)$_3$)$_2$Co(acac)$_3$] (2Pr)

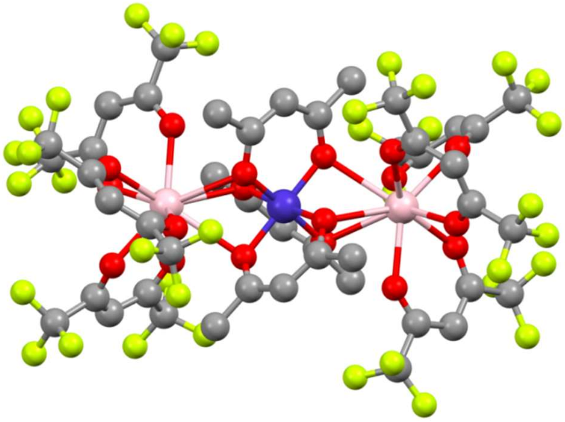

[(Sm(hfac)$_3$)$_2$Co(acac)$_3$] (2Sm)

Figure S11. Optimized structures of [La(hfac)$_3$Ru(acac)$_3$] and [(La(hfac)$_3$)$_2$Ru(acac)$_3$] Cyan, green, grey red and dark green violet spheres are La, F, C, O and Ru atoms, respectively. H atoms are omitted for clarity.

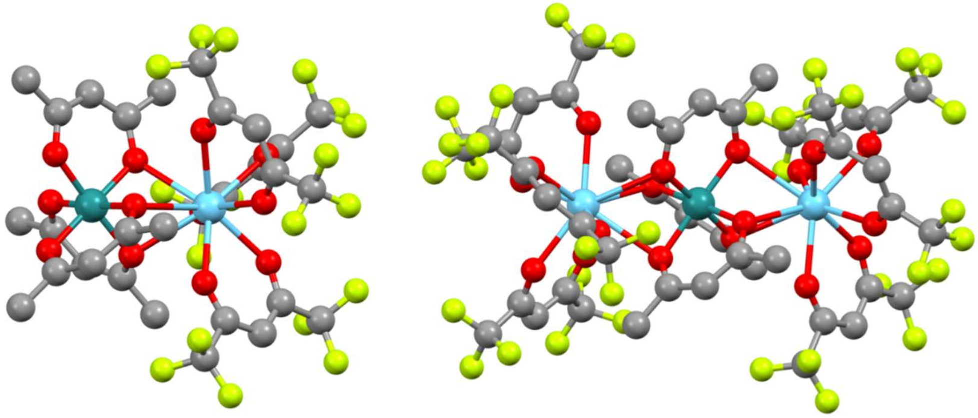

Table S4: Bond lengths in Å and MBO for La-O$_{bridge}$ and M-O$_{bridge}$ of [La(hfac)$_3$M(acac)$_3$] and [(Ln(hfac)$_3$)$_2$M(acac)$_3$] complexes (M = Co, Ru).

	**Bond lengths**		**Mayer bond order (MBO)**	
	La-O$_{bridge}$	M-O$_{bridge}$	La-O$_{bridge}$	M-O$_{bridge}$
[Co(acac)$_3$]	//	1.901	//	0.665
[Ru(acac)$_3$]	//	2.007	//	0.682
[La(hfac)$_3$Co(acac)$_3$]	2.701	1.914<br>(0.7%)	0.161	0.530<br>(-20.3%)
[La(hfac)$_3$Ru(acac)$_3$]	2.738	2.024<br>(0.8%)	0.105	0.564<br>(-17.3%)
[(La(hfac)$_3$)$_2$Co(acac)$_3$]	2.759	1.904	0.126	0.587<br>(-11.7%)
[(La(hfac)$_3$)$_2$Ru(acac)$_3$]	2.787	2.013	< 0.1	0.623<br>(-8.7%)

Table S5: Calculated ΔE and ΔG values (kcal/mol) for [La(hfac)$_3$M(acac)$_3$] and [(La(hfac)$_3$)$_2$M(acac)$_3$] complexes (M= Co, Ru).

Product	Reagents	ΔE	ΔG
[La(hfac)$_3$Co(acac)$_3$]	[Co(acac)$_3$] and [La(hfac)$_3$]	-45.2	-28.4
[La(hfac)$_3$Ru(acac)$_3$]	[Ru(acac)$_3$] and [La(hfac)$_3$]	-42.8	-25.7
[(La(hfac)$_3$)$_2$Co(acac)$_3$]	[Co(acac)$_3$] and 2[La(hfac)$_3$]	-81.7	-47.0
[(La(hfac)$_3$)$_2$Ru(acac)$_3$]	[Ru(acac)$_3$] and 2[La(hfac)$_3$]	-78.4	-43.6
[(La(hfac)$_3$)$_2$Co(acac)$_3$]	[La(hfac)$_3$Co(acac)$_3$] and [La(hfac)$_3$]	-36.5	-18.6
[(La(hfac)$_3$)$_2$Ru(acac)$_3$]	[La(hfac)$_3$Ru(acac)$_3$] and [La(hfac)$_3$]	-35.7	-17.9

Table S6: Assignment of the observed IR band to vibrational modes for[La(hfac)$_3$Co(acac)$_3$]

Calculated (cm^{-1}) (Unscaled)	Calculated (cm^{-1}) (Scaled) [a]	Experimental (cm^{-1})	Vibration*
1093.91	1079.69	1098	C-F stretching La fragment
1147.72	1132.80	1141	C-F stretching La fragment
1206.27	1190.59	1196	CH bending La fragment
1268.05	1251.57	1250	H_3C-C stretching Co fragment
1392.74	1374.63	1375	CH_3 bending Co fragment
1488.17	1468,82	1473	CH bending La fragment
1518.23	1498.49	1480	HC-C stretching Co fragment
1533.52	1513.58	1514	HC-C stretching La fragment
1555.03	1534.81	1569	C-O stretching Co fragment
1630.55	1609.35	1650	C-O stretching La fragment

*The majority of harmonic modes were composed of a variety of local modes. Only selected local modes are listed.

[a]Obtained from the wavenumbers calculated at PBE-D3BJ/def2-TZVP using scaling factors 0.987 [7].

Table S7: Assignment of the observed IR band to vibrational modes for [(La(hfac)$_3$)$_2$Co(acac)$_3$]

Calculated cm^{-1} (Unscaled)	Calculated cm^{-1} (Scaled) [a]	Experimental cm^{-1}	Vibration*
1095.65	1081.41	1097	C-F strect frag La
1152.81	1137.82	1137	C-F strect frag La
1206.86	1191.17	1196	Bend CH La frag
1272.90	1256.35	1250	H_3C-C strct frag Co
1374.03	1356.17	1345	Bend CH_3 Co frag
1482.22	1462.95	1462	Bend CH La frag
1533.88	1513.94	1530	HC-C strect on Co frag
1627.35	1606.19	1646	C-O strect on La frag

*The majority of harmonic modes were composed of a variety of local modes. Only selected local modes are listed.

[a]Obtained from the wavenumbers calculated at PBE-D3BJ/def2-TZVP using scaling factors 0.987. [7]

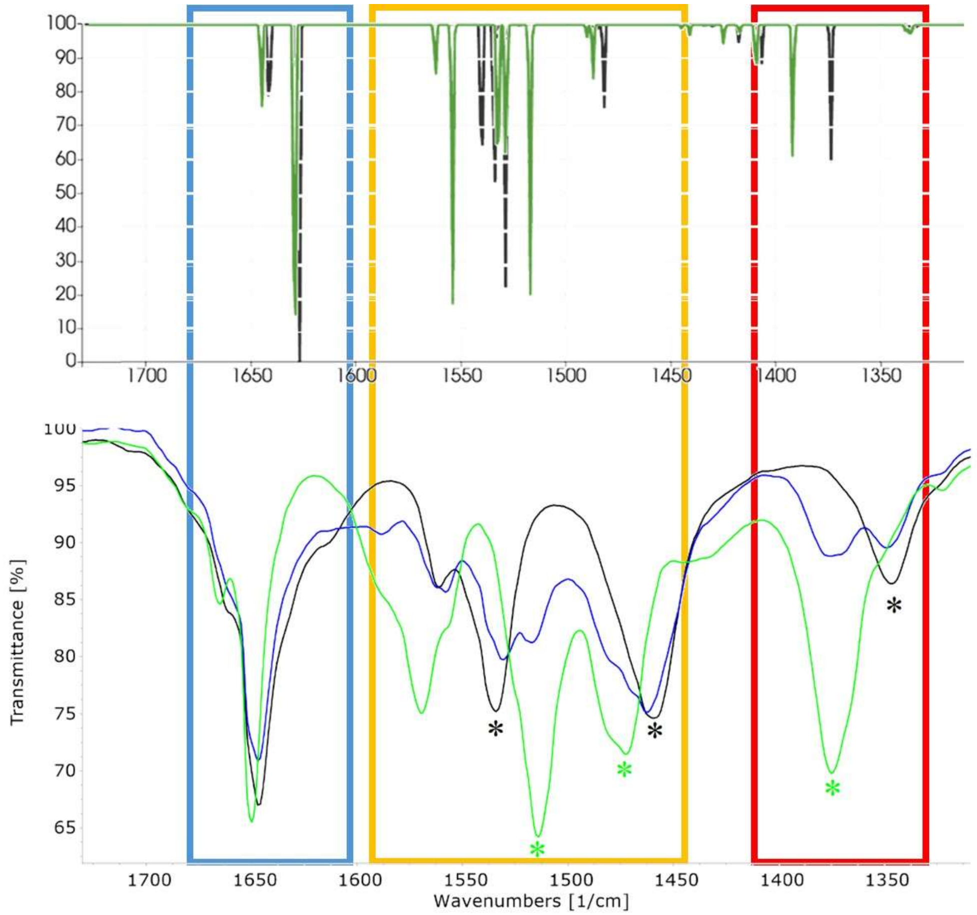

Figure S12: Comparison of calculated and experimental spectra for [La(hfac)$_3$Co(acac)$_3$] (green) and [(La(hfac)$_3$)$_2$Co(acac)$_3$] (black). The three selected colored areas help the reader to better compare the most relevant parts of the IR spectra.

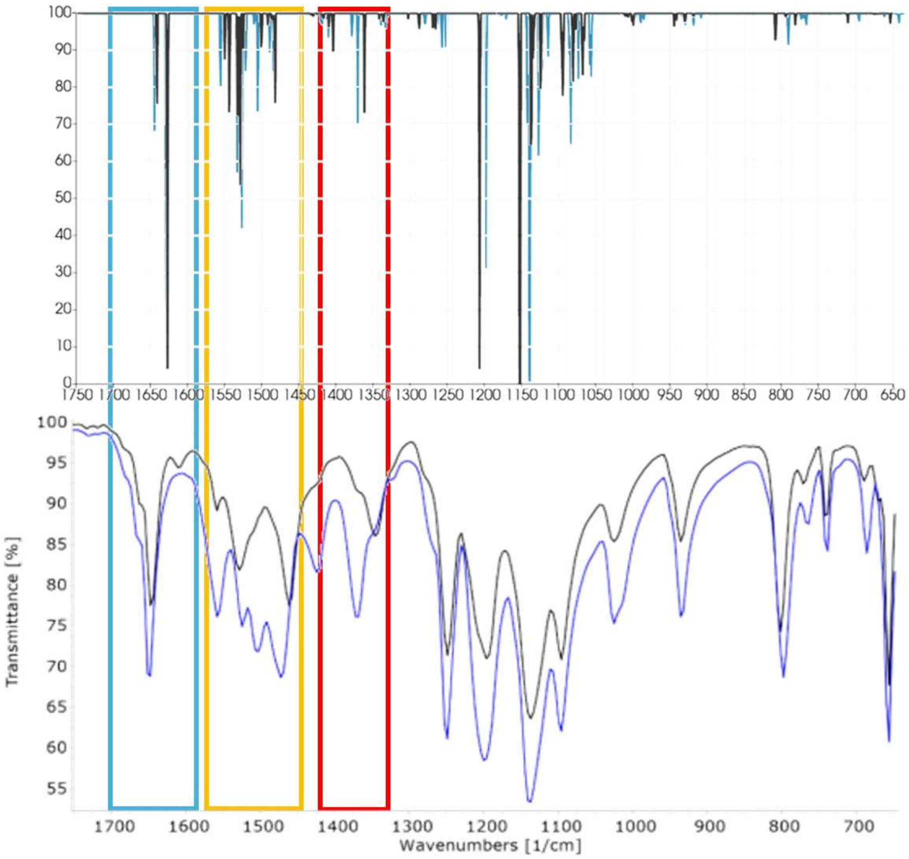

Figure S13: Comparison of calculated and experimental spectra for [La(hfac)$_3$Ru(acac)$_3$] (black) and [(La(hfac)$_3$)$_2$Ru(acac)$_3$] (blue). The three selected colored areas help the reader to better compare the most relevant parts of the IR spectra.

References

[1] Bruker, APEX4 V2021.10-0, Bruker AXS Inc., Madison, Wisconsin, USA, 2021, 2021.

[2] Bruker, SAINT v8.30A, Bruker AXS Inc., Madison, Wisconsin, USA, 2012.

[3] Bruker, XPREP V2014/2, Bruker AXS Inc., Madison, Wisconsin, USA, 2014.

[4] Bruker, SADABS V2016/2, Bruker AXS Inc., Madison, Wisconsin, USA, 2016.

[5] Sheldrick, G.M. SHELXL-2019/1, Bruker AXS Inc., Madison, Wisconsin, USA, 2019.

[6] Lindoy, L. F.; Lip, H. C.; Louie, H.W.; Drew, M.G.B.; Hudson, M.J. Interaction of lanthanide shift reagents with co-ordination complexes; direct observation of nuclear magnetic resonance signals for free and complexed tris(pentane-2,4-dionato)cobalt(III) at ambient temperature, and X-ray crystal and molecular structure. *J. Chem. Soc. Chem. Commun.* **1977**, 778, DOI: 10.1039/c39770000778

[7] Chaabene, M.; Agren, S.; Allouche, A. R.; Lahcinie, M.; Chaabane, R. B.; Baouab, M. H. V. Theoretical and experimental investigations of complexation with $BF_3.Et_2O$ effects on electronic structures, energies and photophysical properties of Anil and tetraphenyl (hydroxyl) imidazole. *Appl Organometal. Chem.* **2019,** *33*, e5218, DOI: 10.1002/aoc.5218**.**
